# Osteoclast indices in osteogenesis imperfecta: systematic review and meta-analysis

**DOI:** 10.1093/jbmrpl/ziae112

**Published:** 2024-08-21

**Authors:** Sirion Aksornthong, Priyesh Patel, Svetlana V Komarova

**Affiliations:** Department of Experimental Surgery, McGill University, Montreal, Quebec H3G 1A4, Canada; Shriners Hospital for Children—Canada, Montreal, Quebec H4A 0A9, Canada; Shriners Hospital for Children—Canada, Montreal, Quebec H4A 0A9, Canada; Faculty of Dental Medicine and Oral Health Sciences, McGill University, Montreal, Quebec H3A 1G1, Canada; Department of Experimental Surgery, McGill University, Montreal, Quebec H3G 1A4, Canada; Shriners Hospital for Children—Canada, Montreal, Quebec H4A 0A9, Canada; Faculty of Dental Medicine and Oral Health Sciences, McGill University, Montreal, Quebec H3A 1G1, Canada; Department of Biomedical Engineering, Faculty of Engineering, University of Alberta, Edmonton, Alberta T6G 1H9, Canada

**Keywords:** osteogenesis imperfecta, brittle bone disease, osteoclast, collagen degradation, patient data, mouse models

## Abstract

Osteogenesis imperfecta (OI) is a rare bone fragility disorder caused by mutations in genes encoding collagen type I or that affect its processing. Alterations in osteoclasts were suggested to contribute to OI pathophysiology. We aimed to systematically identify studies reporting measures of osteoclast formation and function in patients and mouse models of OI, to quantify OI-induced changes. The systematic search of Medline, Ovid, and Web of Science identified 798 unique studies. After screening, we included 23 studies for meta-analysis, reporting osteoclast parameters in 310 patients with OI of 9 different types and 16 studies reporting osteoclast parameters in 406 animals of 11 different OI mouse models. The standardized mean difference with 95% confidence interval (CI) was used as the effect size, and random-effects meta-analysis was performed. In patients with OI, collagen degradation markers were significantly higher compared with age-matched controls, with an effect size of 1.23 (CI: 0.36, 2.10]. Collagen degradation markers were the most elevated in the 3- to 7-year-old age group and in patients with more severe forms of OI. Bone histomorphometry demonstrated the trends for higher osteoclast numbers (1.16; CI: −0.22, 2.55) and osteoclast surface (0.43; CI: −0.63, 1.49), and significantly higher eroded surface (3.24; CI: 0.51, 5.96) compared with age-matched controls. In OI mice, meta-analysis demonstrated significant increases in collagen degradation markers (1.59; CI: 1.07, 2.11), in osteoclast numbers (0.94; CI: 0.50, 1.39), osteoclast surface (0.73; CI: 0.22, 1.23), and eroded surface (1.31; CI: 0.54, 2.08). The largest differences were in OI mice with the mutations in *Col1a1* and *Col1a2* genes. There were no differences between males and females in clinical or animal studies. Quantitative estimates of changes in osteoclast indices and their variance for patients with OI are important for planning future studies. We confirmed that similar changes are observed in mice with OI, supporting their translational utility.

## Graphical Abstract

**Graphical Abstract:**
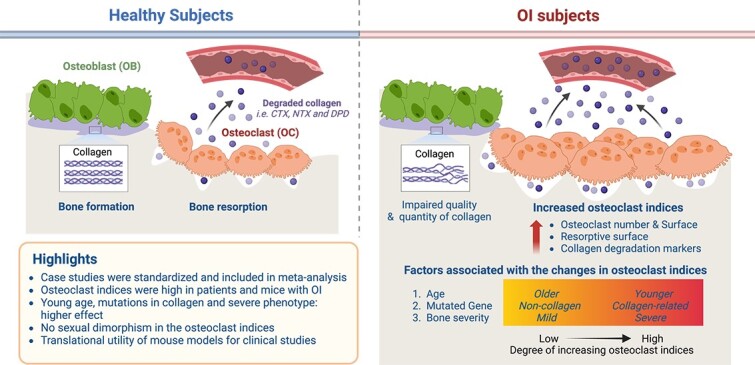

## Introduction

Osteogenesis imperfecta (OI) is a genetic disease with a prevalence of 1 in 10 000–20 000 births, which is characterized by bone fragility, bone deformities, and pain.^1–4^ The majority (85%–90%) of OI cases are caused by the mutation of the genes *Col1a1* or *Col1a2* encoding for collagen type I, the major component in bone extracellular matrix secreted by osteoblasts. Mutations in genes related to the modification, assembly, and vesicular transport of collagen also lead to OI of different severity.^5^ Clinical presentations of OI are classified as nondeforming type I, severe perinatal type II, progressively deforming type III, moderate type IV, and OI with calcification of interosseous membranes and/or hypertrophic callus type V.^6^ The classification of OI types based on the combination of clinical phenotype and the causative mutation, described in the Online Mendelian Inheritance of Man (OMIM) database (http://www.ncbi.nlm.nih.gov/omim/), is now proposed.^4^^,^^6^^,^^7^ Thus, OI is a complex disease with various causative gene mutations and degrees of severity, necessitating different healthcare requirements.

Bone health depends on the function of bone-forming osteoblasts and bone-resorbing osteoclasts. Bone tissue is produced by osteoblasts that secrete large amounts of collagen type I and other components of extracellular matrix and regulate matrix mineralization. Osteoclasts are specialized macrophages responsible for bone degradation. In OI, osteoblasts are unable to produce a functional bone matrix, resulting in the formation of hyper-mineralized, brittle bone tissue.^4^^,^^8^ In addition, bone resorption is higher in individuals with OI compared with their healthy counterparts, resulting in lower bone mass.^9^ Both poor bone tissue properties and low bone mass contribute to reduced bone mechanical properties and a high risk of bone fracture in patients with OI.^4^^,^^10^ Even though collagen is not expressed by osteoclasts, alterations in osteoclast physiology were reported, including increased osteoclast number and size in patients with OI,^11^^,^^12^ and an increased number of osteoclast precursors, osteoclast formation, and function in animal models of OI.^13^^,^^14^ Based on these observations, osteoclast-targeting drugs of the bisphosphonate family were introduced in OI and became standard treatment to suppress bone resorption, leading to improved bone mass and reduced fracture risk in patients with OI.^15^ Thus, osteoclasts are established as significant contributors to OI pathophysiology; however, it is unclear if osteoclast function is similarly affected in different phenotype and severity presentations.

The primary objective of this study was to systematically identify all of the studies that reported osteoclast formation and function in patients and mouse models of OI and to use meta-analysis to quantify the alteration of osteoclast formation and function in patients and mouse models of OI compared with healthy subjects. The secondary objectives were to investigate if osteoclast alterations are associated with different mutations underlying OI and its severity, as well as the age and sex of subjects with OI.

## Materials and methods

### Information sources, search strategy, eligibility criteria, and screening

This study complies with the Preferred Reporting Items for Systematic Reviews and Meta-Analysis (PRISMA) statement (see Supplemental Table 1 for PRISMA checklist) We developed a search strategy that combined keywords and Medical Subject Heading (MeSH) terms for OI (also known as brittle bone disease, fragilitas ossium, and Vrolik’s disease) with those of osteoclast (Supplemental Method 1). No language and time restrictions were applied. The search was performed in Medline, Embase, and Web of Science on April 26, 2021, and the updated search on December 28, 2022. The inclusion criteria were as follows: studies describing patients or experimental animals with OI, which also reported any measures related to osteoclast precursors, differentiation, activity, or survival. We excluded conference abstracts, review articles, secondary studies, editorial commentaries, perspectives/opinions, and patent applications. We also excluded studies reporting only in vitro data or computational models. Studies describing patient treatment were only included if they reported the values preceding the treatment. Screening was performed independently by 2 reviewers (S.A. and P.P.) using Rayyan Systematic Review Screening Software; the disagreements were resolved by discussion between the 2 screeners.

### Data extraction, data items, and conversion

From the full-text articles we extracted the data describing study characteristics and osteoclast parameters. The following data items relevant to study characteristics were extracted (Supplemental Table 2): (1) for all studies: authors; publication year, and country; (2) for studies involving human subjects: publication type (case report or clinical study), age, sex, OI type (when given), OI severity (when given), sample size, control groups (when given), type of diagnosis (clinical or genetic or family history), treatments (when given), and study design; and (3) for animal studies using mouse models of OI: strain, genotype, age, sex, OI severity (when given), sample size, control groups, and treatment (when given). For the osteoclast-related outcomes, we extracted the data for OI and control groups for urine or serum levels of collagen degradation markers, CTx, NTx, or urinary deoxypyridinoline (uDPD),^16^ and osteoclast parameters from bone histomorphometric analysis (osteoclast number, osteoclast surface, resorptive surface, and eroded surface).^17^ Data items included bone type and bone region being measured, type of bone turnover marker, measurement technique, mean or median (as applicable), and standard error (SE), standard deviation (SD), and/or interquartile range (IQR) of reported outcomes. If the type of dispersion measure was not given, we assumed it to be a standard error. If a range of sample sizes was reported, the smallest value was extracted. If only an interval of the age of the subject was provided, the mean value was used. If the studies reported data as a range of minimum, median, and maximum with sample size, the mean (*M*) and SD were estimated^18^ as $\mathrm{M}=\frac{maximum+2 median+ minimum}{4},$$\mathrm{SD}=\sqrt{\frac{maximum- minimum}{4}}$. If the studies reported data as quartile (Q) 1 (Q1), Q2, and Q3 with sample size, the mean and SD were estimated^18^ as $\mathrm{M}=\frac{Q1+Q2+Q3}{3}$, $\mathrm{SD}=\frac{Q3-Q1}{n(n)}$ when *n*(*n*) is correction estimator.^19^ Several studies reported data for healthy controls as mean and SD without reporting sample size,^20–22^ for which we assumed the minimal most conservative sample size as *n* = 3 participants. One study^23^ reported histomorphometric parameters with *n* as the number of fields of histology analyzed, rather than number of patients, in which we also assumed *n* = 3 participants. For collagen degradation markers in patients with OI, several case reports did not state the data for the comparative control group. We performed an additional search to identify the studies reporting normative data for the specific markers, which were also published in the same period using techniques similar to those used in studies reporting OI patient data. From these studies, we extracted the information for the age- and sex-matched control group.

### Study-level outcomes and variance

The standardized mean difference^24^ was calculated as follows:

For the studies reporting aggregated data from OI and control subjects (healthy participants or WT mice), we used the reported means and SDs for OI (*M*_*OI, SDOI*)_ and controls (*M_C,_ SD_C_*) and numbers of subjects in OI (*n_OI_*) and control (*n_C_*) groups to calculate the effect sizes as standardized mean difference $d=\frac{\Delta }{S_p}$, where the raw mean difference ($\Delta )$ was *M_OI_* – *M_C_*, and the pooled SD (*Sp*): $Sp=\frac{\sqrt{\left({n}_{OI}-1\right){SD}_{OI}^2+\left({n}_C-1\right){SD}_C^2}}{n_{OI}+{n}_C-2}$. The standard error for the effect size estimate (SE) was calculated as $SE=\sqrt{\frac{\left({n}_{OI}+{n}_C\right)}{n_{OI}{n}_C}+\frac{d^2}{2\left({n}_{OI}+{n}_C\right)}}$.
For studies presenting aggregated data from patients with OI but failing to report normative data, first we identified the reference control group. If the reference studies were cited, we extracted the normative data from the cited studies. If no reference data were cited, we identified normative data for the same collagen degradation marker measured using the same technique in healthy participants, who were age- and sex- matched to reported patients with OI. The effect sizes were computed as in step 1.
If the study reported the same parameter for several (*k*) OI groups (eg, different age groups), the effect sizes *d_A1_*, *d_A2_*, ..., *d_Ak_* and their SEs *SE_A1_*, *SE_A2_*, ..., *SE_Ak_* were computed separately for each group as in steps 1 and 2 and combined as weighted means to compute a single study-level outcome (*d_com_*, *SE_dcom_*):
  (1) \begin{align*}& {d}_{com}=\frac{\left({n}_{A1}{d}_{A1}+{n}_{A2}{d}_{A2}+\dots +{n}_{Ak}{d}_{Ak}\right)}{n_{A1}+{n}_{A2}+\dots +{n}_{Ak}},\nonumber\\& {SE}_{Dcom}=\frac{\sqrt{\left({n}_{A1}-1\right){SE}_{A1}^2+\left({n}_{A2}-1\right){SE}_{A2}^2+\dots +\left({n}_{Ak}-1\right){SE}_{Ak}^2\ }}{n_{A1}+{n}_{A2}+\dots +{n}_{Ak}-k.} \end{align*}
For studies reporting individual patient data (IPD) for a single or multiple patients, we estimated the standard error for the IPD. First, all the IPD and aggregated data were separated by the type of reported marker. If there were only the IPD in the subset, we combined the IPD for all *k* participants (*x_1_, x_2_, ..., x_k_*) as unweighted means (*M_IPD_*) and computed SD for IPD data within the subset (*SD_IPD_*):
  (2) \begin{align*}& {M}_{IPD}=\frac{\left({x}_1+{x}_2+\dots +{x}_k\right)}{k},\nonumber\\&{SD}_{IPD}=\sqrt{\frac{\left(\sum_{i=1}^k{\left({x}_i-{M}_{IPD}\right)}^2\right)\ }{k}} \end{align*}
  If IPD and aggregated data were presented in the subset, we calculate *SD_IPD_* for IPD for *k* participants, and combined it with *SD_OI_* for *p* groups containing *n_1_*, *n_2_*, ..., *n_p_* patients to estimate the weighted *SD_IPDP_* as follows:
  (3) \begin{align*}& {SD}_{IPD P}=\nonumber\\& \frac{\sqrt{\left(k-1\right){SD}_{IPD}^2+\left({n}_1-1\right){SD}_1^2+\left({n}_2-1\right){SD}_2^2+\dots +\left({n}_p-1\right){SD}_p^2\ }}{k+{n}_1+{n}_2+\dots +{n}_p-p} \end{align*}
  *SD_IPD_* or *SD_IPDP_* values were then assigned to each instance of IPD. The effect size for each patient *i* (*d_i_*) compared with the age-matched control and standard error of effect size (*SE_i_*) were calculated as in step 1 or step 2. Finally, for each study containing *q* patients, we computed a single study-level outcome (*dcom*, *SEdcom*) by combining participants’ *d_i_* and *SE_i_*, as follows:
  (4) \begin{align*} {d}_{com}=\frac{\left({d}_1+{d}_2+\dots +{d}_q\right)}{q},\ \ {SE}_{dcom}=\sqrt{\frac{\left(\sum_{i=1}^n{\left({d}_i-{d}_{com}\right)}^2\right)\ }{q}} \end{align*}
For studies reporting more than 1 collagen degradation marker for the same patient or patient population, we first calculated the effect sizes *d_Mi_*, and *SE_Mi_* for each marker and then combined them as a study-level outcome:
  (5) \begin{align*} {d}_{com}=\frac{\left({d}_{M1}+{d}_{M2}\right)}{2},{SE}_{pM}=\sqrt{\frac{\left(\sum_{i=1}^2{\left({d}_{Mi}-{d}_{com}\right)}^2\right)\ }{2}} \end{align*}
One study reported T-scores for individual patients, which we assumed to be an approximation of the standardized mean difference *d_i_* for each patient. We combined all of the T-scores as unweighted means as a single study-level effect size and calculated SE as in step 4.

### Meta-analysis

Meta-analysis was performed using a random effects (RE) model. The overall effect size ($\hat{\mathrm{\theta}})$ was calculated using study-level outcomes ${d}_{comi}$ with their associated ${SE}_{dcomi}$ for *n* studies with DerSimonian-Laird interstudy variance estimator ${\tau}^2$ as follows:


(6) \begin{align*} \hat{\mathrm{\theta}}=\frac{\sum_i^N\left({d}_{comi}\ \mathrm{x}\ {w}_i\right)}{\sum_i^N{w}_i},{w}_i=\frac{1}{{SE_{dcom}}^2+{\tau}^2} \end{align*}

Standard error of overall effect size was calculated as $SE(\hat{\mathrm{\theta}})=\frac{1}{\sqrt{\sum_i^N{w}_i}}$. 95% CIs were calculated as 95% $\mathrm{CI}=\hat{\mathrm{\theta}}\pm{z}_{1-\alpha /2}\ x\ se(\hat{\mathrm{\theta}})=\hat{\mathrm{\theta}}\pm 1.96\ x\ se(\hat{\mathrm{\theta}})$.

### Exploration of variation (heterogeneity)

The heterogeneity of global outcomes was reported as *I^2^* using Cochran’s Q as a measure of total variation and calculated as the sum of the weighted squared differences between the study-level means θi, as follows:


(7) \begin{align*} &Q=\sum_i se{\left(\mathrm{\theta} \mathrm{i}\right)}^{-2}\times \left({\left(\mathrm{\theta} \mathrm{i}-\frac{\sum_i se{\left(\mathrm{\theta} \mathrm{i}\right)}^{-2}x\ \mathrm{\theta} \mathrm{i}\ }{\sum_i se{\left(\mathrm{\theta} \mathrm{i}\right)}^{-2}}\ \right)}^2\right);\nonumber\\&{H}^2=\frac{Q}{N-1},\mathrm{and}\ {I}^2=\frac{H^2-1}{H^2} \end{align*}

### Study-level risk-of-bias assessment

Quality assessment was performed separately for clinical and animal studies using a 17-question quality checklist (Supplemental Method 2), in which each question was scored as 0, 0.5, or 1. Both checklists included questions on 3 domains: (1) patient or model description, (2) description of methods and results, and (3) presentation quality and alignment. The patient or animal model domain contained 5 questions in the clinical studies checklist and 4 in animal studies, addressing the quality of description of OI type, severity, type of diagnosis, age, sex, treatment, type, description of control, and reporting of ethics approval. The description of methods and results was assessed with 7 questions in the clinical studies checklist and 8 in animal studies addressing the study design, sample acquisition and analysis, and statistical methods. Presentation quality and alignment were assessed with 5 questions in both checklists addressing the quality of tables and graphs; quality and consistency in defining abbreviations, symbols, and units; as well as the nature of the journal (peer-reviewed or not) and the alignment of the study purpose with that of this meta-analysis.

### Risk of bias across studies

Reporting bias was examined using a funnel plot, a meta-regression of reported outcomes with quality score, linear regression of standard error with quality score, single-study exclusion analysis, and cumulative study exclusion analysis.^24^ For single-study exclusion analysis, each study-level outcome was removed 1 at a time and heterogeneity statistics were recalculated. For cumulative study exclusion, the study-level outcomes were excluded sequentially, starting with the most heterogeneous, and heterogeneity statistics were recalculated. Influential studies were analyzed using standardized residual (rstudent), Cook’s distances (cook.d), leave-one-out amount of heterogeneity (tau2.del), and covariance ratio (cov.r).^25–27^

### Additional analysis of covariates

To study the contribution of covariates, we first generated the datasets at the intra-study (IS) level, which shares the common covariate, such as age, disease severity (mild, moderate, other, and not reported [NR]), sex of the subject (male, female, mixed group of both sexes, and NR), and type of collagen degradation marker (sCTx, uDPD, and others). The effect of age was examined using linear regression and Pearson’s correlation, and the data were also grouped as 4 categories: infant, children, adolescent , and adult. The effect size (D_IS_) and the standard error (SE_IS_) were calculated as follows:

For studies reporting aggregated data from only 1 OI population, $d$ and $SE$ were considered as D_IS_ and SE_IS_.
For studies describing multiple groups of patients, D_IS_ and SE_IS_ were calculated as $d$ and $SE$ for each subgroup.
For studies described multiple IPD, $d$ and $SE$ for each IPD were considered as D_IS_ and SE_IS_.

Subgroup analysis was performed by first combining D_IS_ and SE_IS_ reporting the same covariate within the same study for IPD outcomes, then combined D_IS_ (${cD}_{IS}$), and combined SE_IS_ (${cSE}_{IS}$) were calculated as ${cD}_{IS}=\frac{\left({n}_1{D}_{IS1}+{n}_2{D}_{IS2}+\dots +{n}_y{D}_{IS y}\right)}{n_1+{n}_2+\dots +{n}_y}$, ${cSE}_{IS}=\sqrt{\frac{\left(\sum_{i=1}^n{\left({D}_{IS i}-{cD}_{IS}\right)}^2\right)\ }{n_1+{n}_2+\dots +{n}_y}}$, where ${D}_{ISi}$ were the IS study-level effect sizes and ${n}_i$ the number of subjects included in each IS level outcome. Next, ${cD}_{IS}$ and ${cSE}_{IS}$ among different studies that reported the same covariates were grouped together and as the dataset for subgroup analysis, which was performed using the R model and DerSimonian-Laird t estimator. The heterogeneity of each subgroup was presented as *I^2^* and ${\tau}^2.$ The effect of each covariate was also assessed using ANOVA with Tukey’s multiple comparisons.

### Outcome reporting

Effect size is reported as a standardized mean difference ES between OI and age-matched healthy subjects with lower and upper limits of 95% CI as ES (lower CI, upper CI).

### Software

Endnote 20 and Rayyan were used for reference management. WebPlot digitizer was used for data extraction from figures. Microsoft Excel (version 16.16.27) was used for data management and initial calculations. R-studio (version 2023.03.0 + 386) with the metafor package^25^ was used for global outcome, subgroup analysis, and heterogeneity calculations. Figure preparation was accomplished using R-studio, Inkscape (version 1.2), and PRISM (version 9.0.0).

## Results

### Search and screening

**Figure 1:**
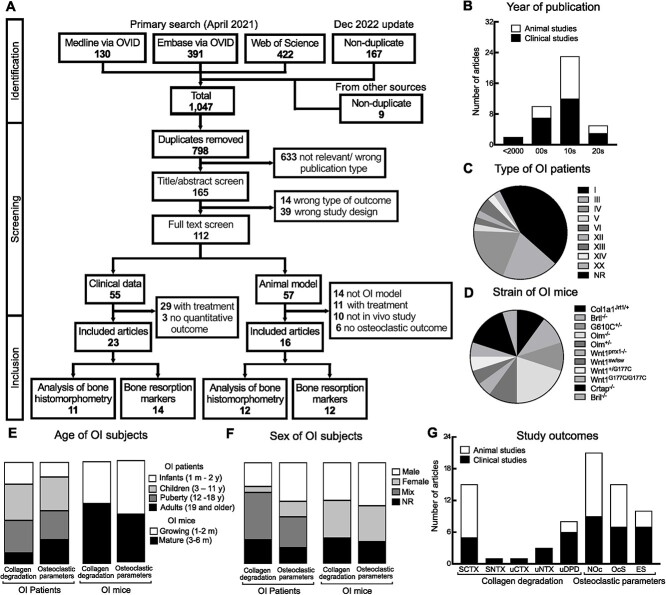
Systematic review and meta-analysis information flow and analysis of studies. (A) PRISMA (Preferred Reporting Items for Systematic reviews and Meta-Analyses) diagram indicates the number of records assessed in each step of the systematic review and meta-analysis. (B) Number of included studies by publication decade. (C, D) Pie charts representing relative contribution to the complete dataset of osteogenesis imperfecta (OI) types reported in clinical studies (C) and OI mouse models (D). (E, F) Reporting of age (E) and sex (F) for collagen degradation markers and histological osteoclast parameters in patients with OI and OI mice. (G) The size of datasets reporting individual osteoclast-related parameters in clinical (black) and animal (white) studies.

The systematic search of Medline, Ovid, and Web of Science identified 798 unique studies (Figure 1A). Title/abstract screening resulted in the inclusion of 165 papers describing studies in patients with OI or OI animal models. After the full-text screening, we identified 112 papers describing osteoclast-related studies in OI, 55 for human participants and 57 for mouse models. For meta-analysis, we included the studies that (1) described untreated subjects, provided the baseline before the treatment, or reported outcomes after treatment termination (excluding 29 human and 11 mouse studies) and (2) provided quantitative estimates of osteoclast-related parameters at the organism level (excluding 3 human and 6 mouse studies that only provided qualitative osteoclast analysis and 10 mouse studies that only reported osteoclast parameters in vitro). Finally, 14 mouse studies were excluded because the mutation was not validated as leading to an OI phenotype. As a result, we included 23 studies published between 1983 and 2021 (Figure 1B, Supplemental Table 3) that reported osteoclast parameters in patients with OI^11^^,^^20–23^^,^^28–45^ and 16 studies reporting osteoclast parameters in mouse models of OI.^13^^,^^46–60^

### Analysis of included studies

We performed a quality assessment of selected studies using the 17-item questionnaires (Method S2). Clinical studies obtained scores between 12 and 15, with the majority of studies scoring between 12 and 13. The common problems identified in clinical studies were poor description and analysis of patient demographics and of the control population. Animal studies obtained scores between 13 and 16, with the majority of studies scoring at 14-15. The common problems identified in animal studies were poor reporting of experimenter blinding for analysis and sample size justification. Thus, the overall quality of studies included in the meta-analysis was good.

Population analysis of included studies demonstrated that, for clinical studies, data for 9 types of OI were reported (Figure 1C, Table 1). The majority of data were for patients with OI with the dominant mutation in collagen type I–encoding genes: type I (13 studies), III (8 studies), and IV (8 studies). Other reports were for patients with OI with recessive mutations in collagen-processing genes and mutations in non–collagen-related genes, with 1 study each for types V, VI, XII, XIV, and XX and 2 studies for type XIII. Three articles reported the osteoclast-related parameter in patients with OI without specifying OI type. For animal studies, we identified 11 strains of OI mice (Figure 1D, Table 2), with 6 studies reporting OI mice with *Col1a2* mutation (*G610C*^*+/−*^*, Oim*^*−/−*^*, Oim*^*+/−*^), 3 studies in OI mice with *Col1a1* mutation (*Col1a1*^*Jrt1/+*^*, Brtl*^*−/−*^), 3 studies in mice with *Wnt1* mutation (*Wnt1*^*prrx1−/−*^*, Wnt1*^*sw/sw*^*, Wnt1*^*+/G177C*^*, Wnt1*^*G177C/G177C*^), and 4 studies reporting data for mice with other OI-associated mutations (*Crtap*^*−/−*^*, Bril*^*−/−*^). Clinical studies reported data for patients with OI with the range of age between 1 month to 42 years, with most cases for the pediatric population, equally distributed between infant (1 month-2 years), child (3-11 years), adolescent (12-18 years), and adult (≥19 years) groups (Figure 1E). In mouse studies, approximately half of the studies reported data for mice younger than 8 weeks old, and the rest for mice aged 9-16 weeks old (Figure 1E). Sex was poorly reported in human studies, with many studies lacking the information for the sex of participants or reporting data for a mixed group of males and females (Figure 1F). In animal studies, a similar proportion of data for male and female mice was reported, and in a notable number of papers animal sex was not reported (Figure 1F).

**Table 1:** Overview of clinical studies included in meta-analysis.

**First author (year)**	**Study design**	**OI type**	**Age, y**	**Sex**	***n***	**CG**	**Markers or parameters**	**Data**	**QS**
**Collagen degradation markers**									
** Pressac (2002)**	CS	I, III, IV	1 mo-17	MF	46	RWS	uCTx1	AG	12
** Iwamoto (2002)**	CR	I	58	M	1	AAR	uNTx, uDPD	IPD	14
** Zacharin (2002)**	CS	III, IV	1-14	MF	18	AAR	uDPD	AG	12.5
** Zacharin (2004)**	CH	Mild	2-15	NR	18	AAR	uDPD	AG	12.5
** Kitaoka (2011)**	CS	I, IV, I/IV	9 mo-13	MF	13	AAR	sNTx	AG	12.5
** Asharani (2012)**	CS	XIII	1-5	NR	2	RWS	uDPD	IPD	12
** D’Eufemia (2014)**	CS	I	2-6	MF	18	AAR	sCTx1	AG	12
** Hryhorovskyi (2015)**	CS	I, III	5-17	NR	4	AMG	sCTx1	IPD, AG	12
** Hoyer-Kuhn (2016)**	CS	I, IV, NA	5-11	MF	10	AAR	sNTx, uDPD	IPD, AG	12
** D’Eufemia (2017)**	CS	I	4-5	MF	18	AAR	sCTx1	AG	13
** Uehara (2017)**	CS	I	14-42	F	3	AAR	uNTx	IPD	12
** Hoyer-Kuhn (2019)**	RT	I, III	8.6	MF	9	AAR	uDPD	AG	12
** Xu (2019)**	CR	XIII	15.7	M	1	RWS	sCTx1	IPD	14
** Zhang (2022)**	CS	I, III, IV, V	14-38	NR	149	RWS	sCTx1	AG	13
**Bone histomorphometric parameters**									
** Baron (1983)**	CH	NR	6-15	MF	9	AMG	NOc, OcS	AG	12.5
** Ste-Marie (1984)**	CS	NR	5-69	MF	12	CAR	NOc, ES	IPD, AG	13
** Rauch (2000)**	CH	I, III, IV	3-11	MF	70	CAR	NOc, OcS, ES	AG	13
** Iwamoto (2002)**	CR	I	58	M	1	AAR	NOc, OcS, ES	IPD	14
** Rauch (2002)**	CS	I, III, IV	1-17	MF	44	CAR	OcS, ES	AG	12.5
** Hryhorovskyi (2015)**	CS	I, III	5-17	NR	3	AAR	NOc, OcS	IPD AG	12
** Ward (2016)**	CR	VI	34 wk	M	1	CAR	NOc, OcS/BS	IPD	15
** Webb (2017)**	CR	XIV	15-24	MF	2	RWS	NOc, OcS, ES	IPD	12
** Surowiec (2020)**	CH	I, III, IV,	1.3-23	MF	8	AAR	NOc	AG	12
** Stürznickel (2021)**	CR	XX	22 wk	NR	1	AMG	NOc, ES	IPD	13
** Lui (2022)**	CR	XII	6	M	1	CAR	ES	IPD	12

The study designs of included clinical studies were case report (CR), case series (CS), retrospective (RT)

or cohort (CH). Sex was reported for male (M) and female (F) patients or for groups of male and female

patients (MF). Control group (CG) was reported as follows: (1) as an age-matched group (AMG), (2) reference values were given but the source unknown (reference without source [RWS]), (3) reference to control is cited, age-matched data extracted (cited age-matched reference [CAR]), (4) control data were not given or cited, age-matched reference assigned (assigned age-matched reference [AAR]). Data types were individual patient data (IPD) or aggregated data (AG). Quality score (QS; out of 17 maximum) was assessed using the checklist (Supplementary method 2).

Abbreviations: ES, eroded surface; NOc, osteoclast numbers; NR, not reported; OcS, osteoclast surface; OI, osteogenesis imperfecta; sCTx, serum CTx; sNTx, serum NTx; uCTx, urinary CTx, uDPD, urinary deoxypyridinoline; uNTx, urinary NTx.

**Table 2:** Overview of animal studies included in meta-analysis.

**First author (year)**	**Genotype**	**Type**	**Age, wk**	**Sex**	***n***	**Markers or parameters**	**Site**	**QS**
**Collagen degradation markers**								
** Kalajzic (2002)**	*Oim^−/−^*	III	4, 12, 20	NR	4-15	uDPD	U	13
	*Oim^+/−^*	IV	–	–	–	–	–	–
** Uveges (2008)**	*Brtl ^−/−^*	II	9, 26	M	7-8	uDPD	U	13
** Chen (2014)**	*Col1a1^Jrt/+^*	IV	5, 20	NR	4	CTx1	S	14
** Grafe (2014)**	*Crtap^−/−^*	VII	8, 16	F	7-14	CTx1	S	15
** Oestreich (2016)**	*Oim^+/−^*	IV	18	NR	15	CTx1	S	14
** Matthews (2017)**	*Oim^−/−^*	III	8	MF	8	CTx1	S	14
** Patoine (2017)**	*Bril^−/−^*	V	6, 12	M	4	CTx1	S	15
** Boraschi-Diaz (2017)**	*Col1a1^Jrt/+^*	IV	16	MF	4-8	CTx1	S	15
** Jeong (2018)**	*Col1a2^G610C+/−^*	IV	18	MF	7-9	CTx1	S	14
	*Oim^−/−^*	III						
** Zimmerman (2018)**	*Oim^−/−^*	III	12	NR	6	CTx1	S	13
** Greene (2021)**	*Col1a2^G610C+/−^*	IV	20	F	10	CTx1	S	15
** Vollersen (2021)**	*Wnt1^+/G177C^*	XV	12	MF	4-5	CTX1	S	15
	*Wnt1^G177C/G177C^*	XV	–	–	–	–	–	–
**Bone histomorphometric parameters**								
** Morello (2006)**	*Ctrap^−/−^*	VII	12	NR	6	NOc, OcS	F	15
** Uveges (2008)**	*Brtl^−/−^*	II	8, 24	M	8-9	NOc, OcS	F	13
** Grafe (2014)**	*Crtap^−/−^*	VII	16	F	6	NOc	LV	15
** Chen (2014)**	*Col1a1^Jrt/+^*	V	8	NR	6	NOc, OcS	F	14
** Joeng (2014)**	*Wnt1^sw/sw^*	XV	6	NR	5	NOc, OcS	LV	16
** Grafe (2016)**	*Ctrap^−/−^*	VII	7, 12	F	6	NOc, OcS	LV	15
** Patoine (2017)**	*Bril^−/−^*	V	9	M	4	NOc, OcS, ES	F	15
** Zimmerman (2018)**	*Oim^−/−^*	III	12	M	8	NOc, OcS	LV	13
** Jeong (2018)**	*G610C^+/−^*	IV	16	MF	3, 4	NOc, OcS	Fr	14
	*Oim^−/−^*	III	–	–	–	–	–	–
** Wang (2019)**	*Wnt1^prrx1−/−^*	XV	6, 12	NR	6	NOc, ES	T	14
** Greene (2021)**	*G610C^+/−^*	IV	10	F	5	NOc	Fr	15
** Vollersen (2021)**	*Wnt1^+/G177C^*	XV	4, 12, 24	MF	3-8	NOc	Fr	15
	*Wnt1^G177C/G177C^*							

The genotype, represented OI type, and age of mice were extracted from the included animal studies. Sex was reported for male (M) and female (F) animals. The site for sample collection was from serum (S), urine (U), femur (Fr), tibia (T), or lumbar vertebrae (LV). Quality score (QS; out of 17 max) was assessed using the checklist (Supplementary Method 2).

Abbreviations: uDPD, urinary deoxypyridinoline; ES, eroded surface; NOc, osteoclast numbers; NR, not reported; OcS, osteoclast surface; OI, osteogenesis imperfecta.

Both clinical (Table 1) and preclinical (Table 2) studies reported collagen degradation markers (14 human and 12 mouse studies) and osteoclast parameters obtained from bone histomorphometry (11 human and 12 mouse studies) (Figure 1G). For the collagen degradation marker**,** serum CTx was commonly used in clinical studies (6 articles) and animal studies (10 articles), followed by uDPD for 6 articles of clinical studies and 2 articles of animal studies. In addition, the concentration of NTx in serum and urinary CTx1 was also reported in clinical studies. The outcomes of the histomorphometric analysis were number of osteoclasts (N.Oc/BS; n/mm), osteoclast surface (Oc.S/BS; %), and eroded surface (ES/BS; %). The iliac crest was the main area of bone biopsy for histomorphometric study in patients with OI. For animal studies, this analysis was performed in the femur (7 articles), lumbar vertebra (4 articles), and tibia (1 article) of OI mice.

Most of the clinical data were reported in case reports, followed by case series and observational cohort studies (Table 1). Clinical studies reported collagen degradation markers for 310 patients with OI with study sample size ranging from 1 to 149 patients. Aggregated data (AG) were reported in 8 studies, IPD in 4 studies, and both AG data and IPD in 2 studies. The comparison was performed to the age-matched group (AMG) in 1 study and reference value without source (RWS) in 4 studies. Nine studies did not report the control group; thus, an assigned age-matched reference (AAR) was applied. Bone histomorphometry analysis was reported for 152 patients with OI, with study sample sizes ranging from 1 to 70 patients. Aggregated data were reported in 4 studies, IPD in 5 studies, and both AG data and IPD in 2 studies. The normal values from healthy control group were reported as cited age-matched reference for 5 studies. The RWS, AMG, and AAR were reported in 1, 2, and 3 studies, respectively. In OI mice, collagen degradation markers were mainly reported in *Oim*^*−/−*^ mice (4 studies), followed by *Oim*^*+/−*^ and *Col1a1*^*Jrt/+*^ mice (2 studies each). Bone histomorphometric analysis was reported in *Ctrap*^*−/−*^ mice (3 studies), followed by *G610C*^*+/−*^ and *Oim*^*−/−*^ mice (2 studies). The age-matched littermates or WT mice were reported as a control group in all animal studies.

### Collagen degradation markers in patients with OI

**Figure 2:**
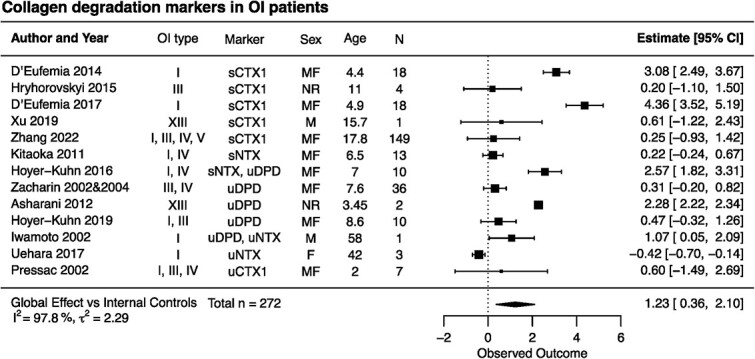
Forest plot of the differences in collagen degradation markers between patients with osteogenesis imperfecta (OI) and healthy controls. Indicated are the included studies; the OI type for reported patients; markers measured in the study; sex of patients as male (M), female (F), or mixed group of male and female (MF) and not reported (NR); average age of patients; and the number of patients. The standardized mean differences with 95% CIs for individual studies are depicted as squares/lines; the square size is proportional to the study’s weight. Diamonds/bands represent the global effect size and CI. A positive difference reflects higher values in OI. The heterogeneity statistics *I^2^* and τ^2^ are reported.

First, we performed the meta-analysis of collagen degradation markers in human participants. We calculated the standardized mean difference between patient and control data for each report. We identified 14 studies, in which there were 5 datasets with sCTx, 2 with sNTx, 6 with uDPD, 2 with uNTx. and 1 with uCTx. Nine studies reported data for OI populations,^29^^,^^32–36^^,^^38^^,^^39^^,^^41^ but not the corresponding control values. In these cases, we identified studies reporting normative data that were cited in the primary publication, or if no reference data were cited, we identified normative data for the same collagen degradation marker measured using the same technique. For studies reporting uDPD in pediatric patients,^32^^,^^33^^,^^36^^,^^41^ the normative data were from Shaw et al,^61^ and for uDPD in adult patients^29^ from Chan et al.^62^ For studies reporting sCTx,^35^^,^^38^ the normative data were from Rauchenzauner et al^63^; for sNTx^34^^,^^36^ from van der Sluis et al^64^; and for uNTx^29^^,^^39^ from Sone et al.^65^ Since the levels of collagen degradation markers show a strong age dependence, we extracted data from the reference publications that matched the age and sex of reported patients with OI. For studies reporting aggregate data, the standardized mean difference was calculated compared with age-matched, within-study control populations^23^^,^^45^ or compared with reference populations providing the best match for age and sex of reported patients with OI.^32^^,^^33^^,^^35^^,^^38^^,^^41^ In 2 studies,^38^^,^^45^ the data for 2 populations of different ages were reported separately and were combined as unweighted means. Four studies^21^^,^^34^^,^^36^^,^^39^ reported IPD for multiple patients, and 3 studies^22^^,^^23^^,^^29^ reported single-patient data. For each of these studies, we calculated the effect size as follows. First, we calculated the mean and SD within IPD studies with multiple patients, then we calculated the SD_m_ for each marker by combining the SD from all the studies reporting the same marker. SD_m_ was then assigned to each IPD value, including those from case reports, and the effect size for each patient compared with the age-matched control or reference value was calculated. Finally, the average within-study effect size was calculated as the unweighted mean of all patients. Two studies^29^^,^^36^ reported 2 markers for the same patient or patient population, for which the effect sizes were combined as unweighted average. One study^30^ reported a T-score for individual patients, which we assumed to be an approximation of the standardized mean difference, and calculated the overall effect size with the CI for the study. Next, we combined the resulting standardized mean differences in the random-effects meta-analysis model, which demonstrated that collagen degradation markers were significantly higher in patients with OI compared with the age-matched control population, with an effect size of 1.23 (CI: 0.36, 2.10) (Figure 2). The heterogeneity was high, with *I^2^* = 97.8% and ${\tau}^2$ = 2.29.

**Figure 3:**
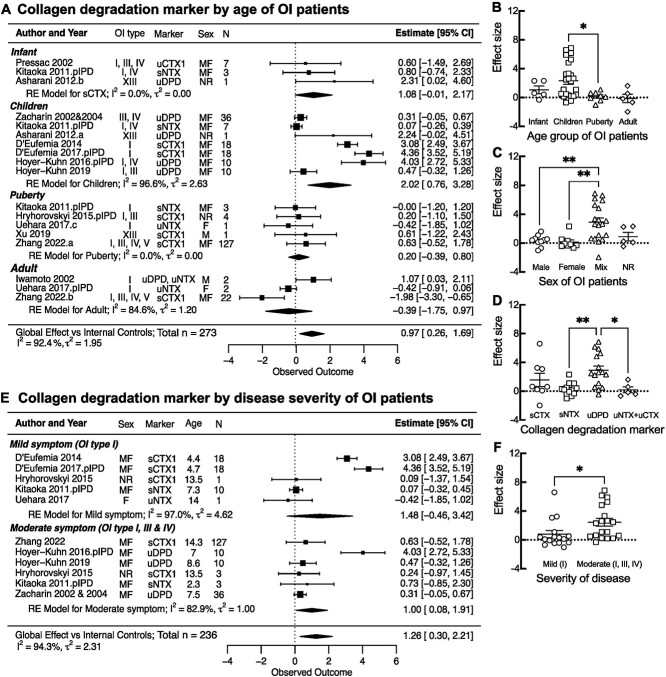
Effect of biological factors on the collagen degradation markers in patients with osteogenesis imperfecta (OI). (A, E) Forest plots of subgroup analysis by age of patients with OI (A) grouped as infants (0–2 years), children (3–11 years), adolescents (12–18 years), and adults (>19 years) and (E) by disease severity groups as mild symptoms (only OI type I) and moderate symptoms (OI type I, III, and IV). Indicated are the included studies; the OI type for reported patients for markers measured in the study; sex of patients as male (M), female (F), mixed group of male and female (MF), and not reported (NR); average age of patients; and the number of patients. The standardized mean differences with 95% CIs for individual studies are depicted as squares/lines; the square size is proportional to the study’s weight. Diamonds/bands represent global effect size and CI for the random effects (RE) model. A positive difference reflects higher values in OI. The heterogeneity statistics *I^2^* and τ^2^ are reported. Scatterplots for the effect sizes from individual outcomes (aggregated or individual patient data [IPD]) are reported in each study, separated by patient age group (B), reported sex (C), reported markers (D), and disease severity (F). Shown are means ± SEMs; ^*^p < .05 and ^*^^*^p < .01 indicate significance difference by 1-way ANOVA with Tukey’s post-test for B, C, and D, or Student’s *t* test for F.

Next, we examined the contribution of different covariates to the observed increase in collagen degradation markers in OI. In the articles describing multiple individual patients or groups of patients, we separated the datasets according to the covariate being tested and combined within-study data with the same covariate as the patient number weighted average. We observed a significant effect of age on the effect size, which was the highest for children aged 3-11 years old (Figure 3A). We also plotted all the individual data points and examined the differences by ANOVA (Figure 3B), which similarly demonstrated significantly higher collagen degradation markers in 3- to 11-year-old children. The effect size was similar in male and female patients in the studies where sex was reported; however, it was higher in the studies that reported mixed groups of patients (Figure 3C, Figure S1). Importantly, the studies that reported the sex of participants enrolled similar numbers of infants, children, adolescents, and adults, while those that reported patients of both sexes together were mostly for 3–11-year-old children, suggesting that the effect size of the mixed group was higher because it mostly consisted of children. Among the markers of bone resorption, uDPD demonstrated the highest increases (Figure 3D, Figure S2); however, the data were mostly from children (3-11 years old). Finally, to examine the effect of disease severity on bone degradation markers, we used only data from pediatric populations (up to 15 years old). We separated the data in 2 groups: (1) patients with mild-type I OI and (2) and patients with moderate to severe OI (OI type II, III, or IV reported separately, or OI type I, III, or IV reported as a group). Collagen degradation markers were significantly higher in the more severely affected patients (Figure 3E, F). Thus, our data suggest a strong contribution of age and severity to the degree of change in collagen degradation markers.

### Bone histology in patients with OI

We identified 11 studies that reported the bone histomorphometric analysis from the iliac, transiliac, and transcortical iliac crest biopsies of patients with OI. There were 9 studies reporting osteoclast numbers/bone surface or per bone perimeter (N.Oc/BS, N.Oc/B.Per, 1/mm)^11^^,^^23^^,^^28^^,^^29^^,^^37^^,^^40^^,^^42^^,^^43^ or osteoclast numbers per bone area (N.Oc/BA, 1/mm^2^)^20^; 7 datasets reported osteoclast surface/bone surface (OcS/BS, %)^11^^,^^28^^,^^29^^,^^31^^,^^37^^,^^40^^,^^44^ and 7 datasets reported eroded surface per bone surface (ES/BS, %).^11^^,^^20^^,^^29^^,^^31^^,^^40^^,^^43^^,^^44^ Two studies reported the internal control as the age- and sex-matched group (AMG),^24^^,^^43^ 5 studies reported the cited age-matched reference,^11^^,^^25^^,^^28^^,^^36^^,^^44^ 1 study reported data for the OI population and RWS,^39^ and 3 studies used the AAR.^26^^,^^34^^,^^42^

When reference normative data were cited, we identified the studies and extracted the age-matched normative data from the control group. The normative data from Glorieux et al^66^ were the most used as a reference control in OI studies,^20^^,^^29^^,^^31^^,^^37^^,^^40^^,^^44^ which we also used for the study that did not cite the reference control. Bone histomorphometric parameters were reported as AG data in 6 studies,^11^^,^^20^^,^^23^^,^^28^^,^^31^^,^^42^ for which the standardized mean difference was calculated compared with age-matched control populations. From 7 studies reporting IPD,^20^^,^^23^^,^^29^^,^^37^^,^^40^^,^^43^^,^^44^ 3 studies provided data for multiple patients and 4 for a single patient. For studies reporting multiple IPD,^20^^,^^23^^,^^40^ we first calculated the mean and SD for each study, then calculated the SD_m_ for each parameter by combining SD from all the studies reporting the same parameter. SD_m_ was assigned to IPD, including the 4 case reports.^29^^,^^37^^,^^43^^,^^44^ Next, we computed the effect size for each IDP compared to the reference control population. For each study, the average within-study effect size was calculated as the unweighted mean of all patients. The study-level outcomes were combined in the random-effects, meta-analysis model.

**Figure 4:**
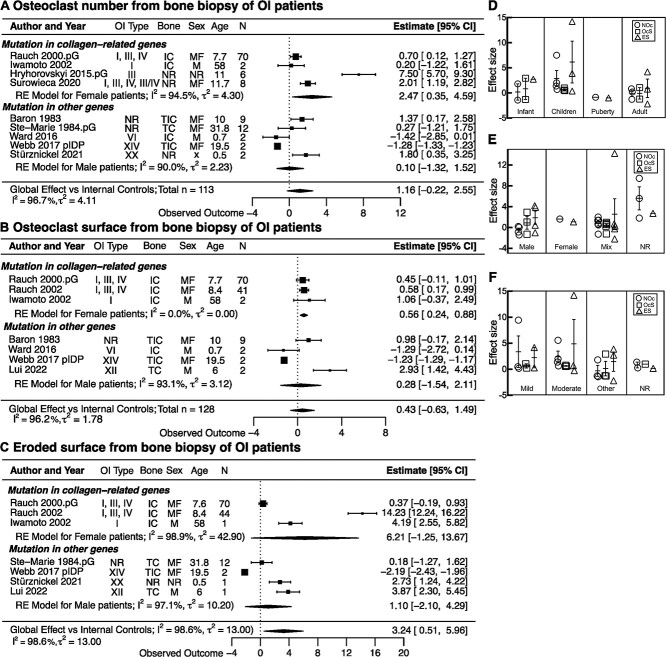
The differences in histomorphometric osteoclast parameters between patients with osteogenesis imperfecta (OI) and healthy controls. (A–C) Forest plot of the differences in osteoclast numbers (A), osteoclast surface (B), and eroded surface (C) between patients with OI and healthy controls. Indicated are the included studies; the OI type for reported patients; site of bone biopsy (transiliac crest [TC], iliac crest [IC], and transcortical iliac crest [TIC]); sex of patients as male (M), female (F), mixed group of male and female (MF), and not reported (NR); average age of patients; and the number of patients. The standardized mean differences with 95% CIs for individual studies are depicted as squares/lines; the square size is proportional to the study’s weight. Diamonds/bands are global effect sizes and CI. A positive difference reflects higher values in OI. The heterogeneity statistics *I^2^* and τ^2^ are reported. (D–F) The effect sizes from individual outcomes for osteoclast numbers (NOc; circles), osteoclast surface (OcS; squares), or eroded surface (ES; triangles) reported in each study as aggregated data or individual patient data (IPD) were separated by patient age (D), reported sex (E), and disease severity (F). Shown are means ± SEM; no statistical significance by 1-way ANOVA. Abbreviation: RE, random effects.

Meta-analysis of bone histomorphometry outcomes demonstrated the trends for higher osteoclast number in 113 patients with OI, with an effect size of 1.16 (CI: −0.22, 2.55), and osteoclast surface in 128 patients with OI, with an effect size of 0.43 (CI: −0.63, 1.49), as well as significantly higher eroded surface in 131 patients with OI, with an effect size of 3.24 (CI: 0.51, 5.96) compared with the age-matched control population (Figure 4A–C). The heterogeneity indices were high for all 3 datasets: *I^2^* > 90%, ${\tau}^2$ = 2-13. When we separated the data by the type of the underlying mutation in patients with OI, it was evident that osteoclast number and surface were significantly increased in patients with a mutation in collagen genes, OI type I, III, and IV, with an effect size for osteoclast number of 2.47 (CI: 0.35, 4.59) and for osteoclast surface of 0.56 (CI: 0.24, 0.88). In contrast, in patients with OI type VI, XIV, and XX, osteoclast number and surface were not significantly affected, with respective effect sizes of 0.1 (CI: −1.32, 1.52) and 0.28 (CI: −1.54, 2.11). The eroded surface was similarly affected in both patients with OI with mutations in collagen (type I, III, and IV) and with mutations with collagen-processing genes (type VI, XIV, and XX). When we analyzed the effect of covariates on bone histomorphometric markers, there was no difference in the level of effect size for osteoclast-related parameters (osteoclast number, surface, eroded surface) among groups of different ages (Figure 4D), sex (Figure 4E), or disease severity (Figure 4F), although the effect size representing osteoclast number and eroded surface tended to increase in children compared with other age groups.

### Collagen degradation markers in OI mice

**Figure 5:**
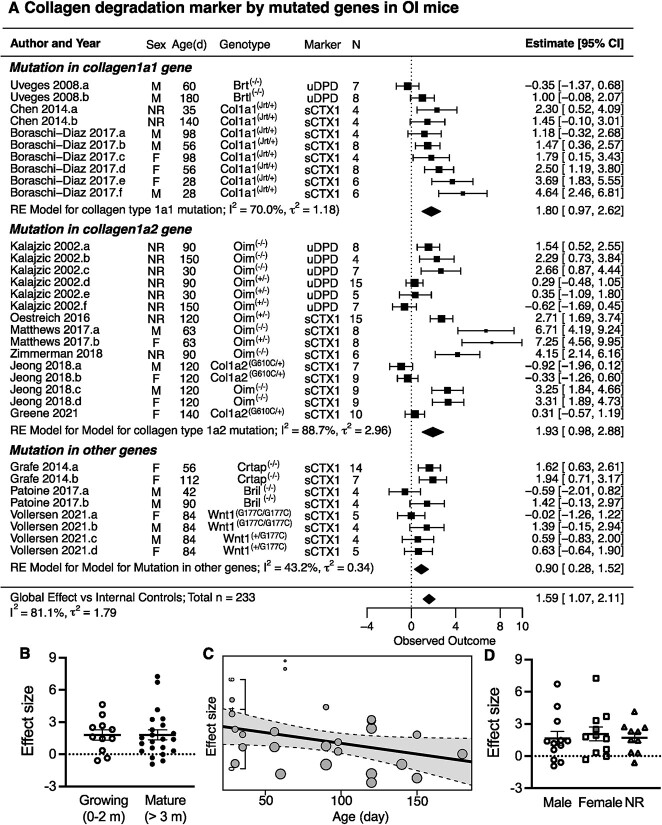
The differences in collagen degradation markers between osteogenesis imperfecta (OI) mice and healthy controls. (A) Forest plot of the differences in collagen degradation markers between OI mice grouped based on the underlying mutation in the *Col1a1* gene, *Col1a2* gene, or other genes and corresponding controls. Indicated are the included studies; animal sex (male [M], female [F], or not reported [NR]); age and genotype; markers measured in the study; and sample size. The standardized mean differences with 95% CIs for individual studies are depicted as squares/lines; the square size is proportional to the study’s weight. Diamonds/bands are group and global effect sizes and CI. A positive difference reflects higher values in OI. The heterogeneity statistics *I^2^* and τ^2^ are reported. (B) Individual effect sizes in growing (younger than 2 months) and mature (older than 3 months) mice. (C) Meta-regression analysis of age effect on the effect size in OI mice: *R^2^* = 0.1163, adjusted *R^2^* = 0.07787. (D) Individual effect sizes for male and female OI mice, and in studies that did not report the sex (NR). For B and D, shown are means ± SEM; no statistical significance by 1-way ANOVA. Abbreviation: RE, random effects.

We identified 12 studies reporting collagen degradation markers in mouse models of OI. All of the studies presented data from groups of OI and the age-matched littermates or WT control mice, with a sample size of 4-15 mice per group. The standardized mean difference between OI and control mice was calculated as the study-level effect size. A single study effect size was computed for each of the 3 studies reporting 1 group of OI mice.^52^^,^^57^^,^^59^ For the studies that reported the experiments with multiple groups of OI mice of different ages,^13^^,^^46^^,^^49^^,^^53^^,^^55^ sexes,^53^^,^^54^^,^^56^^,^^60^ and genotypes,^46^^,^^54^^,^^56^^,^^60^ the effect size was computed for each group of OI mice with the corresponding control group. Taken together, the dataset included 33 groups with a total of 233 OI mice. The overall effect size for collagen degradation markers was 1.59 (CI: 1.07, 2.11), with high heterogeneity (*I^2^* = 81.1% and ${\tau}^2$ = 1.79) (Figure 5). Collagen degradation markers sCTx1 and uDPD were increased to a higher degree in OI mice with collagen mutations, with an overall effect size of 1.80 (CI: 0.97, 2.62) for OI mouse models with mutations in *Col1a1* and 1.93 (CI: 0.98, 2.88) for OI mice with mutations in *Col1a2*, compared with OI mice with mutations in other genes 0.90 (CI: 0.28, 1.52) (Figure 5A). There was no difference in collagen degradation markers between the growing and mature OI mice (Figure 5B). A mild negative correlation between the effect size and the age of OI mice identified in meta-regression (*R^2^* = 0.12) may indicate higher bone resorption in younger OI mice (Figure 5C). No significant difference associated with animal sex was observed (Figure 5D).

### Bone histology in OI mice

We identified 12 studies that reported the bone histomorphometric analysis from the tibia, femur, and lumbar vertebra of OI mice. There were 12 studies reporting osteoclast numbers/bone surface or per bone perimeter (N.Oc/BS, N.Oc/B.Per; 1/mm)^47–51^^,^^55–58^^,^^60^ or osteoclast numbers per bone area (N.Oc/BA; 1/mm^2^).^13^^,^^59^ Eight studies reported osteoclast surface/bone surface (OcS/BS; %)^13^^,^^46^^,^^47^^,^^50^^,^^51^^,^^55–57^ and 2 reported eroded surface per bone surface (ES/BS; %).^55^^,^^58^ All of the studies presented data from groups of OI and WT mice with a sample size of 3–9 animals per group. The standardized mean difference between OI and control mice was calculated as the study-level effect size separately for each parameter of histomorphometric analysis. A single study-level effect size was computed for each of the outcomes for the studies reporting data for osteoclast number, osteoclast surface, and eroded surface for 1 group of OI mice.^47–50^^,^^55^^,^^57^^,^^59^ When a study reported multiple groups of OI mice of different ages,^13^^,^^49^^,^^58^^,^^60^ sexes,^56^^,^^60^ and genotypes,^56^^,^^60^ the effect size was computed for each outcome for each group of OI mice relative to the corresponding control group.

**Figure 6:**
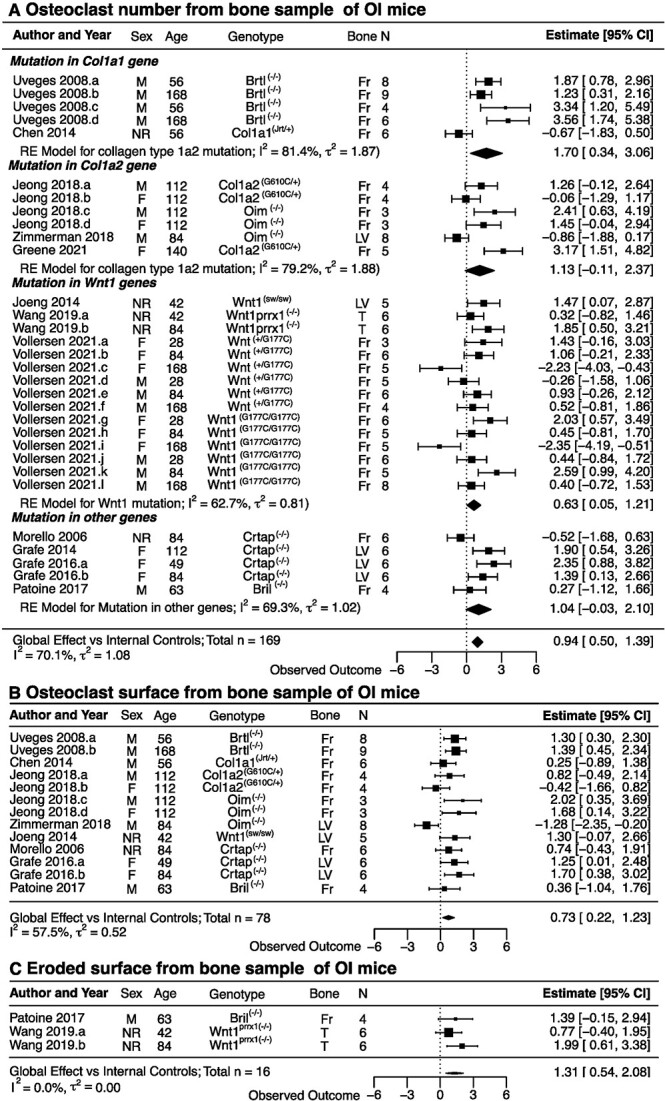
The differences in histomorphometric osteoclast parameters between osteogenesis imperfecta (OI) mice and healthy controls. (A–C) Forest plots of the differences in osteoclast numbers (A), osteoclast surface (B), and eroded surface (C) between OI mice and healthy controls. Indicated are the included studies; animal sex (male [M], female [F], or not reported [NR]); age and genotype; bone analyzed (femur [F], lumbar vertebrae [LV], or tibia [T]); and sample size. The standardized mean differences with 95% CIs for individual studies are depicted as squares/lines; the square size is proportional to the study’s weight. Diamonds/bands are global effect sizes and CI. A positive difference reflects higher values in OI. The heterogeneity statistics *I*^2^ and τ^2^ are reported. Abbreviation: RE, random effects.

Meta-analysis of bone histomorphometric outcomes demonstrated the significantly increased osteoclast number in 169 OI mice compared with age-matched WT mice, with an effect size of 0.94 (CI: 0.50, 1.39) (Figure 6A) and increased osteoclast surface in 78 OI mice compared with age-matched WT mice with an effect size of 0.73 (CI: 0.22, 1.23) (Figure 6B) and eroded surface in 16 OI mice compared with age-matched WT mice with an effect size of 1.31 (CI: 0.54, 2.08) (Figure 6C). The heterogeneity was moderate for osteoclast number and surface (*I^2^* = 58%–70%, ${\tau}^2$ = 0.5-1) and low for eroded surface (*I^2^* = 0%, ${\tau}^2$ = 0). Subgroup analysis based on the type of the underlying mutation in OI mice showed significantly increased osteoclast numbers in OI mice with *Co1a1* mutations, with an effect size of 1.70 (CI: 0.34, 3.06), and with *Wnt1* mutations, with an effect size of 0.63 (CI: 0.05, 1.21) (Figure 6A). No difference in osteoclast number, osteoclast surface, or eroded surface was found when OI mice with mutations in *Co1a1*, *Co1a2*, *Wnt1*, or other genes were compared by ANOVA (Figure S3A). Similarly, no significant difference was identified in osteoclast number, osteoclast surface, or eroded surface between growing and skeletally mature mice (Figure S3B), or male and female mice (Figure S3C). Linear regression demonstrated no correlation between osteoclast number or osteoclast surface and the age of mice (Figure S3D, E). While the eroded surface strongly correlated with age (Figure S3F), the number of mice with this outcome was low.

### Publication bias, heterogeneity, and sensitivity analyses

To analyze the publication bias, effect of study quality, and the sources of heterogeneity, we used the largest datasets in clinical and animal studies, collagen degradation markers in OI subjects (Figure S4). The funnel plots of clinical (Figure S4A) and animal (Figure S4J) datasets indicated a lack of funnel-like structure, consistent with low sample sizes of individual studies and a corresponding absence of high-precision studies. The asymmetry of the funnel plot was evident for the clinical dataset. Meta-regression demonstrated no significant correlation between the quality of clinical or animal studies (Figure S4B, K) and the effect size. Linear regression demonstrated no significant correlation between the publication quality and reported standard error (Figure S4C, L). Single-study exclusion analysis identified 1 influential study in the clinical dataset^23^ (Figure S4D), but not in the animal dataset (Figure S4M). Cumulative study exclusion demonstrated that 73% of studies in the clinical dataset and 22% in the animal dataset needed to be removed to obtain a homogenous dataset (Figure S4E, N). We also examined if a single study significantly influenced the heterogeneity of the dataset using standardized residual (rstudent) (Figure S4F, O), Cook’s distances (cook.d) (Figure S4G, P), leave-one-out amount of heterogeneity (tau2.del) (Figure S4H, Q), and covariance ratio (cov.r) (Figure S4I, R).^25–27^ While, in the clinical dataset, no single study significantly affected heterogeneity indices, in the animal dataset 1 study^54^ significantly influenced the heterogeneity of the dataset.

## Discussion

We performed a systematic review and meta-analysis to assess how much and how consistently osteoclast biology and function are affected in patients with OI and animal models of OI. We demonstrated that collagen degradation markers were significantly increased in patients with OI compared with age-matched healthy control subjects and in mice with mutations leading to OI-like phenotype compared with their healthy littermates. Analysis of bone histomorphometry data demonstrated increased eroded surface in bone biopsy samples of patients with OI and an increase in osteoclast number, osteoclast surface, and eroded surface in bone samples of OI mice. We observed a strong association of age with osteoclast markers in patients with OI, with the highest levels reported in the patient group aged 3 to 7 years. There were no differences between males and females in osteoclast function in OI subjects from clinical or animal studies. The indices of osteoclast function were higher in patients with OI with more severe presentations compared with those with mild disease, and patients with OI and OI mice with mutations in *Col1a* and *Col1a2* genes compared with those with mutations in other genes. Our study demonstrates a significantly higher osteoclast function in patients with OI and OI mice, of which the highest degree of osteoclast dysfunction was present in young patients with more severe disease due to mutations in collagen type I.

The pathogenesis of brittle bone is well known to be involved with the abnormal collagen production by osteoblasts, leading to a reduction in the quantity of functional collagen fiber, abnormal collagen structure, and hyper-mineralization of bone matrix, which results in the impaired mechanical properties of the bone and increased fracture risk in patients with OI.^67–69^ Moreover, OI is also commonly characterized by low bone mass,^9^^,^^70^^,^^71^ which has been attributed to the abnormal function of osteoclasts.^13^^,^^14^^,^^72^ Although collagen is not expressed by osteoclasts, osteoclastogenesis was shown to be modulated by collagen receptors, such as osteoclast-associated receptor (OSCAR), which binds collagen and stimulates osteoclastogenesis,^73^ and leukocyte-associated immunoglobulin-like receptor 1 LAIR-1 receptor that provides a negative feedback for osteoclast formation.^74^ The OI collagen type I differs from healthy collagen type I in amino acid sequence due to the underlying mutation and additional post-translational modifications of OI collagen,^75–77^ which cause a cascade of changes in collagen processing and mineralization.^67^^,^^68^ However, less is known about the direct effect of the mutant collagen structure on receptor binding and consequent regulation of osteoclast differentiation and function. With the high heterogeneity among causative gene mutations, phenotype severity, and other biological factors of patients with OI, whether and how osteoclasts contribute to the pathogenesis of brittle bone in OI remains unclear. This meta-analytic study allowed us to quantitatively summarize the alteration in osteoclast physiology and function in OI subjects.

Bisphosphonates, drugs that specifically target osteoclasts, have been successfully used in children with OI to improve bone mass and prevent fractures.^15^^,^^78^^,^^79^ In this study, we only included papers reporting osteoclast indices for patients with OI prior to treatment with bisphosphonate or after the drug holiday to minimize the treatment effect on osteoclast physiology. Osteoclast function in clinical studies can be assessed by bone histomorphometry, which requires invasive transiliac biopsy,^17^ or by examining blood or urine levels of collagen degradation markers indicative of increased bone turnover.^80^ Previously, prominent increases in bone resorption markers were reported in children with OI,^32^^,^^36^^,^^38^ while osteoclast indices in bone histomorphometry were variably affected.^11^^,^^20^ Our study suggests that, in addition to the age of the individual, the underlying mutation affects the degree of change in osteoclast markers, with higher osteoclast function observed in patients with mutations in collagen type I. Moreover, our data suggest that osteoclast indices are higher in patients with more severe disease, indicating the important contribution of osteoclast dysfunction to OI pathophysiology.

We identified 11 strains of OI mice that mimic diverse types of causative gene mutations, including OI mice with *Col1a1* mutation (*Col1a1*^*Jrt1/+*^*, Brtl*^*−/−*^), OI mice with *Col1a2* mutation (*G610C*^*+/−*^*, Oim*^*−/−*^*, Oim*^*+/−*^), *Wnt1* mutation (*Wnt1*^*prrx1−/−*^*, Wnt1*^*sw/sw*^*, Wnt1*^*+/G177C*^*, Wnt1*^*G177C/G177C*^), and other OI-associated mutations (*Crtap*^*−/−*^*, Bril*^*−/−*^). All of the animal studies provided the age-matched control group, which allowed us to detect the increases in collagen degradation markers, osteoclast number and surface, and eroded surface with higher certainty. We analyzed the effects of age, sex, and the type of gene mutation on alteration of osteoclast function. While we did not observe similar trends for the effect of age on collagen degradation markers in mice, it is important to note that only 2 studies examined groups of very young mice, 3-4 weeks old, which are age-equivalent to young children found to have higher collagen degradation markers in clinical studies. Similar to clinical studies, sex did not affect the osteoclast indices in OI mice. Importantly, the higher degree of increases in collagen degradation markers and osteoclast numbers was observed in OI mice with *Col1a1* and *Col1a2* mutations compared with those with other gene mutations, suggesting that abnormal collagen structure might directly affect osteoclast formation and function. Thus, our meta-analysis emphasizes the translational capacity of research performed in OI animal models.

The clinical data included in our study were presented as small studies, case series, and case reports. While case reports are considered to be biased evidence and are commonly excluded,^24^^,^^81^ a large proportion of data for rare diseases are presented as case reports, which are often thorough and detailed, providing valuable clinical information. It was previously suggested that meta-analysis of case reports provides comparable estimates to those of larger studies.^82^ By including case reports, we maximized the use of available data, obtaining the datasets for collagen degradation markers in 310 patients with OI (1 study with 149 patients, 8 studies with 9-46 patients, and 5 studies with 1-4 patients) and for bone histomorphometry in 152 patients (2 studies with 44 and 70 patients, 3 studies with 8-12 patients, and 6 studies with 1-3 patients). This shows significant and underutilized potential for knowledge synthesis studies in rare disorders such as OI.

We have identified several limitations for our study. The first set of limitations was due to the nature of OI as a rare disease with pediatric presentation. The scarcity of data led us to include older studies that did not use contemporary diagnosis tools and did not specify OI type or severity. Thus, it is possible that some patients included in the analysis did not have OI according to current diagnostic criteria. However, most studies (15 of 25) were from 2014-2022, suggesting minimal bias due to the inclusion of patients with unrelated conditions. Moreover, OI clinical studies focused on pediatric population, and very few studies reported data for older patients with OI, limiting the analysis of age-related changes. Mouse models of OI also lack clear criteria for assigning an OI-like phenotype to mice with mutations in genes other than those known to lead to OI in patients (*Col1a1*, *Col1a2*, *Crtap*, *Bril*). Similar to the clinical data, the range of ages reported for OI mice was relatively small; however, for animal studies, we found very few papers studying young mice that mimic the pediatric clinical population. The second set of limitations was due to poor reporting of the patient age, sex, and ethnicity or reporting their findings as groups of patients of mixed age and sex, which limited our ability to ensure that all datasets included in the analysis are independent and presented challenges in analysis of the contribution of underreported parameters. One of the surprising limitations was a severe lack of reporting of normative data in clinical studies of collagen degradation markers. Even though these markers are commonly used in clinical practice, it is important to report the normative means and variance for the current study, since the methodologies (and corresponding normative data) may change with time. We overcame this by using published normative data from the same time period obtained using the same method as that reported in the paper; however, some uncertainty in our estimates remains.

In conclusion, taken together, our study provides quantitative estimates of changes in osteoclast indices and their variance for patients with OI and suggests a novel hypothesis for associations of these changes with mutations in collagen genes and with disease severity. Moreover, combining data from numerous publications on this rare disease allowed us to investigate the effects of covariates on the degree of osteoclast dysfunction in patients with OI, which are important for planning future studies. We confirmed that similar changes are observed in mice with OI, supporting their translational utility. Our study contributes to developing personalized medicine according to gene mutation and age group to finetune osteoclast function to improve bone strength in patients with OI.

## Supplementary Material

Revised_Supplemental_Material_2024_07_25_ziae112

## Data Availability

The datasets used and/or analyzed during the current study are available from the corresponding author on reasonable request.
