# Supplementary material for: Osteoclast indices in osteogenesis imperfecta: systematic review and meta-analysis
Source: JBMR Plus. 2024 Aug 21;8(11):ziae112. doi: 10.1093/jbmrpl/ziae112 (PMC11450326; doi:10.1093/jbmrpl/ziae112)
Supplement: Revised_Supplemental_Material_2024_07_25_ziae112 [file revised_supplemental_material_2024_07_25_ziae112.pdf]

Supplemental Materials

Supporting information for the manuscript "Osteoclast indices in osteogenesis imperfecta: systematic review and meta-analysis" Sirion Aksornthong, Priyesh Patel, Svetlana V. Komarova

Supplemental Method 1: Search strategy

Example of search strategy applied for Web of Science database

1 = (TS = (osteogenesis imperfecta))
2 = (TS =(fragilitas ossium))
3 = (TS =(vrolik disease))
4 = #1 OR #2 OR #3
5 = (TS =(osteoclast*))
6 = (TS =(osteoclastogenesis))
7 = #5 OR #6
8 = #4 AND #7

url: https://www.webofscience.com/wos/woscc/summary/e018209c-afb0-4ec4-92da-3d5919865c98-933dc204/relevance/1

Supplemental Method 2: Quality checklist

S2.1 Description of checklist for quality assessment for clinical study

1) Was the diagnosis of OI patient (clinical/genetic, type, severity) well defined?
2) Was the demographic background of the OI population clearly described?
3) Did the control subjects come from a similar demographic background to the OI patients?
4) Were OI patient treatments well described including medication frequency and timing?
5) Was ethics approval/ informed consent stated in the manuscript?
6) Was the process of OI patients and control selection well described?
7) Did the authors specify all aspects of the process of sample acquisition?
8) Was the method for outcome assessment consistent across all participants?
9) Did the authors perform blind analysis for all outcomes and study groups?
10) Did the authors analyze outcomes from all participants, if not, did they describe how to choose the subset for an assessment?
11) Was the statistical method of analysis clearly described?
12) Was the analysis of the outcome separated by sex?
13) Were data tables and graphs presented in good quality?
14) Were the abbreviations and symbols used in the study well-defined in all the figures and tables?
15)Were the units of assessed outcomes correct and well-defined?
16) Did the article publish in peer review journal?
17) Did the objective of the study align with the meta-analytic project?

S2.2 Description of checklist for quality assessment for animal study

1) Was the OI model clearly described?
2) Was the background described and WT mice matched with the OI (age-match and littermates)?
3) Were the WT and OI animals kept in the same conditions (housing, diet manipulation, treatment regimen)?
4) Was the ethics approval stated in the manuscript?
5) Did the authors clearly describe the frequency/timeframe of sampling/treatment?
6) Was the animal group allocation process well described?
7) Did the authors specify all aspects of the process of sample acquisition?
8) Was the method for outcome assessment consistent across all animals?
9) Did the authors perform blind analysis for all outcomes and study groups?
10) Was the sample size justification, power description, or variance and effect estimates well defined?
11) Was the method of the statistical analysis reported?
12) Was the analysis of the outcome separated by sex?
13) Were data tables and graphs reported with good quality?
14) Were the abbreviations and symbols used in the study well-defined in all the figures and tables?
15) Were the units of assessed outcomes correct and well-defined?

16) Was the article published in a peer-reviewed journal?
17) Was the objective of the study aligned with the meta-analytic project?

Supplemental Table 1: Prisma Checklist

Section and Topic	Item #	Checklist item	Location where item is reported
**TITLE**			
Title	1	Identify the report as a systematic review.	1
**ABSTRACT**			
Abstract	2	See the PRISMA 2020 for Abstracts checklist.	2
**INTRODUCTION**			
Rationale	3	Describe the rationale for the review in the context of existing knowledge.	3
Objectives	4	Provide an explicit statement of the objective(s) or question(s) the review addresses.	3,4
**METHODS**			
Eligibility criteria	5	Specify the inclusion and exclusion criteria for the review and how studies were grouped for the syntheses.	4
Information sources	6	Specify all databases, registers, websites, organisations, reference lists and other sources searched or consulted to identify studies. Specify the date when each source was last searched or consulted.	4
Search strategy	7	Present the full search strategies for all databases, registers and websites, including any filters and limits used.	S2
Selection process	8	Specify the methods used to decide whether a study met the inclusion criteria of the review, including how many reviewers screened each record and each report retrieved, whether they worked independently, and if applicable, details of automation tools used in the process.	4
Data collection process	9	Specify the methods used to collect data from reports, including how many reviewers collected data from each report, whether they worked independently, any processes for obtaining or confirming data from study investigators, and if applicable, details of automation tools used in the process.	4
Data items	10a	List and define all outcomes for which data were sought. Specify whether all results that were compatible with each outcome domain in each study were sought (e.g. for all measures, time points, analyses), and if not, the methods used to decide which results to collect.	4, S4, T1, T2
	10b	List and define all other variables for which data were sought (e.g. participant and intervention characteristics, funding sources). Describe any assumptions made about any missing or unclear information.	4, S4, T1, T2
Study risk of bias assessment	11	Specify the methods used to assess risk of bias in the included studies, including details of the tool(s) used, how many reviewers assessed each study and whether they worked independently, and if applicable, details of automation tools used in the process.	6
Effect measures	12	Specify for each outcome the effect measure(s) (e.g. risk ratio, mean difference) used in the synthesis or presentation of results.	4-5
Synthesis methods	13a	Describe the processes used to decide which studies were eligible for each synthesis (e.g. tabulating the study intervention characteristics and comparing against the planned groups for each synthesis (item #5)).	5
	13b	Describe any methods required to prepare the data for presentation or synthesis, such as handling of missing	4-7

Section and Topic	Item #	Checklist item	Location where item is reported
		summary statistics, or data conversions.	
	13c	Describe any methods used to tabulate or visually display results of individual studies and syntheses.	7
	13d	Describe any methods used to synthesize results and provide a rationale for the choice(s). If meta-analysis was performed, describe the model(s), method(s) to identify the presence and extent of statistical heterogeneity, and software package(s) used.	5
	13e	Describe any methods used to explore possible causes of heterogeneity among study results (e.g. subgroup analysis, meta-regression).	5-7
	13f	Describe any sensitivity analyses conducted to assess robustness of the synthesized results.	5-6
Reporting bias assessment	14	Describe any methods used to assess risk of bias due to missing results in a synthesis (arising from reporting biases).	6
Certainty assessment	15	Describe any methods used to assess certainty (or confidence) in the body of evidence for an outcome.	4
**RESULTS**			
Study selection	16	Describe the results of the search and selection process, from the number of records identified in the search to the number of studies included in the review, ideally using a flow diagram.	Fig1
Study characteristics	17	Cite each included study and present its characteristics.	7, T2, T3
Risk of bias in studies	18	Present assessments of risk of bias for each included study.	T2, T3
Results of individual studies	19	For all outcomes, present, for each study: (a) summary statistics for each group (where appropriate) and (b) an effect estimate and its precision (e.g. confidence/credible interval), ideally using structured tables or plots.	Fig. 2-7
Results of syntheses	20a	For each synthesis, briefly summarise the characteristics and risk of bias among contributing studies.	Fig. 2-7
Results of syntheses<br>Reporting biases	20b	Present results of all statistical syntheses conducted. If meta-analysis was done, present for each the summary estimate and its precision (e.g. confidence/credible interval) and measures of statistical heterogeneity. If comparing groups, describe the direction of the effect.	Fig. 2-7
	20c	Present results of all investigations of possible causes of heterogeneity among study results.	Fig.8
	20d	Present results of all sensitivity analyses conducted to assess the robustness of the synthesized results.	Fig.8
	21	Present assessments of risk of bias due to missing results (arising from reporting biases) for each synthesis assessed.	Fig.8
Certainty of evidence	22	Present assessments of certainty (or confidence) in the body of evidence for each outcome assessed.	Fig.8
**DISCUSSION**			
Discussion	23a	Provide a general interpretation of the results in the context of other evidence.	11-12
	23b	Discuss any limitations of the evidence included in the review.	12
	23c	Discuss any limitations of the review processes used.	12
	23d	Discuss implications of the results for practice, policy, and future research.	13

Supplemental Table 2: Data items extracted from the included clinical and animal studies.

**Parameters**	**Identified data**
Subject species	Human, mouse
Study design	Clinical studies: case series, case report, retrospective and cohort<br>Animal studies: in vivo study in mouse model of OI
Diagnostic approach	Clinical assessment, Radiography, Genetic test and Family history
OI type	I, II, III, IV, V, XII, XIII, XIV, XVI and XX
Strain OI mice	*Col1a1* mutation: $Col1a1^{(Jrt/+)}$, $Brtl^{-/-}$<br>*Col1a2* mutation: $G610C^{+/-}$, $Oim^{-/-}$, $Oim^{+/-}$<br>*Wnt1* mutation: $Wnt1^{+/G177C}$ $Wnt1^{G177C/G177C}$, $Wnt1^{prrx1-/-}$, $Wnt1^{sw/sw}$<br>Other: $Bril^{-/-}$, $Crtap^{-/-}$
Age	Clinical studies: weeks, months, years<br>Animal studies: weeks
Sex	Male, Female, Male and female combined and Not reported
Sample size	Clinical studies: number of patients and controls<br>Animal studies: number of mice/groups
Healthy control	Clinical studies: *i)* age-matched group (AMG), *ii*) reference values were given but the source unknown (reference without source (RWS)), *iii*) reference to control is cited, age-matched data extracted (cited age-matched reference (CAR)), *iv*) control data was not given or cited, age-matched reference assigned (assigned age-matched reference (AAR)).<br>Animal studies: age-matched littermate control (LC) and age-matched wild-type (WT)
Bone resorption markers	Serum markers: CTX1 (ng/mL or pg/mL, or T-score) and NTX (nmolBCE/l)<br>Urinary marker: NTX (nmol BCE/mmol Cr or pmole/umole Cr) and DPD (nM/mM Cr or nmol /mmol Cr or ug/g Cr)
Histomorphometric analysis of osteoclast-related parameters	Number of osteoclast = (N.Oc/BS, N.Oc/B.Per, 1/mm, N.Oc/BA, $1/mm^2$)<br>Osteoclast surface = Oc.S/BS (%)<br>Eroded surface= ES/BS (%)

Parameters described the characteristics of OI subjects, study design, diagnostic approach, type of OI for patients or OI mouse model for animal studies, age, sex, sample size, type of control group, the included bone resorption markers and osteoclast-related parameters from histomorphometric analysis.

Supplemental Table 3: Extraction table

Clinical studies of collagen degradation markers														
			OI subjects						Heathy control subjects					
Author, year	Marker	Method	OI type	Sex	Age (y)	N	Mean	SD	Reference	Sex	Age (y)	N	Mean	SD
D'Eufemia 2014	sCTX	Chemiluminescence (ng/ml)	I	MF	2 - 6	18	6.50	4.50	Rauchenzauner 2007	MF	2.5 - 7.5	150	1.64	0.67
D'Eufemia 2017	sCTX	Chemiluminescence (ng/ml)	I	MF	4 - 5	12	5.22	3.40	Rauchenzauner 2007	MF	2.5 - 7.5	150	1.64	0.67
D'Eufemia 2017	sCTX	Chemiluminescence (ng/ml)	I	MF	4 - 5	6	9.02	5.00	Rauchenzauner 2007	MF	2.5 - 7.5	150	1.64	0.67
Xu 2019	sCTX	Chemiluminescence (ng/ml)	XIII	M	15.7	1	0.97	1.84	AMG	NR	NR	3	0.33	0.06
Zhang 2022	sCTX	Chemiluminescence (ng/ml)	I, III, IV, V	MF	14.3	127	0.65	0.42	AMR	MF	7 – 15	3	0.39	0.25
Zhang 2022	sCTX	Chemiluminescence (ng/ml)	I, III, IV, V	MF	38.3	22	0.13	0.07	AMR	MF	27 – 46	3	0.33	0.24
Hryhorovskyi 2015	sCTX	Chemiluminescence (ng/ml)	I	NR	5 - 17	1	0.69	1.84	AMG	NR	NR	21	0.57	1.31
Hryhorovskyi 2015	sCTX	Chemiluminescence (ng/ml)	III	NR	5 - 17	3	0.87	0.16	AMG	NR	NR	21	0.57	1.31
Kitaoka 2011	sNTX	ELISA (nmolBCE/l)	I	M	7	1	50.50	29.12	van der Sluis 2002	M	7.7	2	41.91	10.12
Kitaoka 2011	sNTX	ELISA (nmolBCE/l)	I	M	4	1	59.00	29.12	van der Sluis 2002	M	8.21	6	56.05	24.77
Kitaoka 2011	sNTX	ELISA (nmolBCE/l)	I	F	10	1	76.50	29.12	van der Sluis 2002	F	10.3	5	64.87	21.17
Kitaoka 2011	sNTX	ELISA (nmolBCE/l)	I	M	12	1	79.00	29.12	van der Sluis 2002	M	12.08	4	58.89	12.93
Kitaoka 2011	sNTX	ELISA (nmolBCE/l)	I	F	4	1	47.20	29.12	van der Sluis 2002	F	8.29	6	53.34	12.96
Kitaoka 2011	sNTX	ELISA (nmolBCE/l)	I	M	4	1	38.00	29.12	van der Sluis 2002	M	8.21	6	56.05	24.77
Kitaoka 2011	sNTX	ELISA (nmolBCE/l)	I	F	4	1	58.50	29.12	van der Sluis 2002	F	8.29	6	53.34	12.96
Kitaoka 2011	sNTX	ELISA (nmolBCE/l)	I	M	14	1	80.00	29.12	van der Sluis 2002	M	14.56	4	110.9	30.66
Kitaoka 2011	sNTX	ELISA (nmolBCE/l)	I	F	13	1	36.60	29.12	van der Sluis 2002	F	13.33	2	38.23	5.72
Kitaoka 2011	sNTX	ELISA (nmolBCE/l)	I	M	1	1	66.60	29.12	van der Sluis 2002	M	8.21	6	56.05	24.77
Kitaoka 2011	sNTX	ELISA (nmolBCE/l)	IV	F	1	1	47.80	29.12	van der Sluis 2002	F	8.29	6	53.34	12.96
Kitaoka 2011	sNTX	ELISA (nmolBCE/l)	IV	M	6	1	46.50	29.12	van der Sluis 2002	M	7.73	2	41.91	10.12
Kitaoka 2011	sNTX	ELISA (nmolBCE/l)	IV	F	0	1	92.00	29.12	van der Sluis 2002	F	8.29	6	53.34	12.96
Hoyer-Kuhn 2016	sNTX	ELISA (nmolBCE/l)	I, IV	MF	5 - 11	10	85.48	39.56	van der Sluis 2002	MF	7 - 11	40	59.45	17.86
Hoyer-Kuhn 2016	uDPD	HPLC (nmol/mmol Cr)	I, IV	MF	5 - 11	1	43.52	31.76	Shaw 1995	MF	5 – 11	81	38.87	25.02

Clinical studies of collagen degradation markers (continue)

Author, year	Marker	Method	OI subjects						Heathy control subjects					
			OI type	Sex	Age (y)	N	Mean	SD	Reference	Sex	Age (y)	N	Mean	SD
Hoyer-Kuhn 2016	uDPD	HPLC (nmol/mmol Cr)	I, IV	MF	5 - 11	1	104.0	31.76	Shaw 1995	MF	5 – 11	81	38.87	25.02
Hoyer-Kuhn 2016	uDPD	HPLC (nmol/mmol Cr)	I, IV	MF	5 - 11	1	105.4	31.76	Shaw 1995	MF	5 – 11	81	38.87	25.02
Hoyer-Kuhn 2016	uDPD	HPLC (nmol/mmol Cr)	I, IV	MF	5 - 11	1	122.4	31.76	Shaw 1995	MF	5 – 11	81	38.87	25.02
Hoyer-Kuhn 2016	uDPD	HPLC (nmol/mmol Cr)	I, IV	MF	5 - 11	1	160.1	31.76	Shaw 1995	MF	5 – 11	81	38.87	25.02
Hoyer-Kuhn 2016	uDPD	HPLC (nmol/mmol Cr)	I, IV	MF	5 - 11	1	170.9	31.76	Shaw 1995	MF	5 – 11	81	38.87	25.02
Hoyer-Kuhn 2016	uDPD	HPLC (nmol/mmol Cr)	I, IV	MF	5 - 11	1	187.0	31.76	Shaw 1995	MF	5 – 11	81	38.87	25.02
Hoyer-Kuhn 2016	uDPD	HPLC (nmol/mmol Cr)	I, IV	MF	5 - 11	1	193.8	31.76	Shaw 1995	MF	5 – 11	81	38.87	25.02
Hoyer-Kuhn 2016	uDPD	HPLC (nmol/mmol Cr)	I, IV	MF	5 - 11	1	211.3	31.76	Shaw 1995	MF	5 – 11	81	38.87	25.02
Hoyer-Kuhn 2016	uDPD	HPLC (nmol/mmol Cr)	I, III	MF	5 - 11	10	48.33	17.97	Shaw 1995	MF	8 – 9	17	40.79	14.64
Zacharin 2004	uDPD	HPLC (nmol/mmol Cr)	mild	NA	2 - 15	18	26.70	13.50	Shaw 1995	MF	2 - 15	110	38.88	27.61
Iwamoto 2002	uDPD	HPLC (nmol/mmol Cr)	I	M	58	1	7.40	31.76	Chan 2004	M	68 ± 7	46	4.85	0.79
Asharani 2012	uDPD	NR (nmol/mmol Cr)	XIII	NA	5	1	58.66	31.76	AMR	NR	NR	3	16.50	5.00
Asharani 2012	uDPD	NR (nmol/mmol Cr)	XIII	NA	1.9	1	63.90	31.76	AMR	NR	NR	3	19.50	7.20
Zacharin 2002	uDPD	HPLC (nmol/mmol Cr)	NR	MF	1 - 1 4	18	66.20	6.70	Shaw 1995	MF	2 - 15	115	38.20	27.42
Uehara 2017	uNTX	ELISA (nmol/mmol Cr)	I	F	42	1	19.30	10.25	Sone 1995	F	40 - 49	43	26.20	10.30
Uehara 2017	uNTX	ELISA (nmol/mmol Cr)	I	F	40	1	24.40	10.25	Sone 1995	F	40 - 49	43	26.20	10.30
Uehara 2017	uNTX	ELISA (nmol/mmol Cr)	I	F	14	1	32.90	10.25	Sone 1995	F	20 - 29	30	37.50	11.10
Iwamoto 2002	uNTX	ELISA(nmol/mmol Cr)	I	M	58	1	42.70	10.25	Sone 1995	M	40 - 49	43	26.20	10.30
Pressac 2002	uCTX	ELISA	I, III, IV	MF	1 - 4	1	T-score = -2.22		AMR	MF	4 - 10			
Pressac 2002	uCTX	ELISA	I, III, IV	MF	1 - 4	1	T-score =-1.47		AMR	MF	4 - 10			
Pressac 2002	uCTX	ELISA	I, III, IV	MF	1 - 4	1	T-score =-0.99		AMR	MF	4 - 10			
Pressac 2002	uCTX	ELISA	I, III, IV	MF	1 - 4	1	T-score =-0.56		AMR	MF	4 - 10			
Pressac 2002	uCTX	ELISA	I, III, IV	MF	1 - 4	1	T-score =0.42		AMR	MF	4 - 10			

Clinical studies of collagen degradation markers (continue)													
Author, year	Marker	Method	OI subjects						Heathy control subjects				
			OI type	Sex	Age (y)	N	Mean	Reference	Sex	Age (y)	N	Mean	SD
Pressac 2002	uCTX	ELISA	I, III, IV	MF	1 - 4	1	T-score =3.79	AMR	MF	4 - 10			
Pressac 2002	uCTX	ELISA	I, III, IV	MF	1 - 4	1	T-score = 5.21	AMR	MF	4 - 10			

**Animal studies of collagen degradation markers**															
Author, year	Marker	Method	Unit	OI mice						Heathy control mice					
				Genotype	Sex	Age (d)	N	Mean	SD	Control mice	Sex	Age (d)	N	Mean	SD
Kalajzic 2002	uDPD	PYRILINKS-D assay	nM/mM Cr	$Oim^{-/-}$	NR	30	7	33.51	4.28	WT	NR	30	3	23.28	2.06
Kalajzic 2002	uDPD	PYRILINKS-D assay	nM/mM Cr	$Oim^{+/-}$	NR	30	5	24.20	2.83	WT	NR	30	3	23.28	2.06
Kalajzic 2002	uDPD	PYRILINKS-D assay	nM/mM Cr	$Oim^{-/-}$	NR	90	8	16.38	4.80	WT	NR	90	12	10.78	2.66
Kalajzic 2002	uDPD	PYRILINKS-D assay	nM/mM Cr	$Oim^{+/-}$	NR	90	15	11.44	1.97	WT	NR	90	12	10.78	2.66
Kalajzic 2002	uDPD	PYRILINKS-D assay	nM/mM Cr	$Oim^{-/-}$	NR	150	4	16.47	3.85	WT	NR	150	7	10.78	1.37
Kalajzic 2002	uDPD	PYRILINKS-D assay	nM/mM Cr	$Oim^{+/-}$	NR	150	7	9.98	1.20	WT	NR	150	7	10.78	1.37
Chen 2014	sCTX	ELISA	ng/mL	$Col1a1^{(Jrt/+)}$	NR	35	4	96.80	27.10	WT littermate	NR	35	4	43.90	17.90
Chen 2014	sCTX	ELISA	ng/mL	$Col1a1^{(Jrt/+)}$	NR	35	4	12.45	3.20	WT littermate	NR	35	4	8.90	1.30
Zimmerman 2018	sCTX	ELISA	ng/ml	$Oim^{-/-}$	NR	90	6	516.20	171.10	WT littermate	NR	90	6	14.25	2.72
Oestreich 2016	sCTX	ELISA	ng/ml	$Oim^{+/-}$	NR	120	15	124.66	37.14	WT littermate	NR	120	13	20.60	39.70
Patoine 2017	sCTX	ELISA	ng/ml	$Bril^{-/-}$	M	42	4	42.58	6.36	WT littermate	M	42	4	47.89	10.96
Patoine 2017	sCTX	ELISA	ng/ml	$Bril^{-/-}$	M	90	4	33.74	4.29	WT littermate	M	90	4	26.95	5.21
Jeong 2018	sCTX	ELISA	ng/ml	$Col1a2^{(+/G610C)}$	M	120	7	10.54	2.51	WT littermate	M	120	9	17.70	10.05
Jeong 2018	sCTX	ELISA	ng/ml	$Oim^{-/-}$	M	120	9	197.94	77.82	WT littermate	M	120	9	17.70	10.05
Jeong 2018	sCTX	ELISA	ng/ml	$Col1a2^{(+/G610C)}$	F	120	9	11.01	5.65	WT littermate	F	120	9	12.52	3.14
Jeong 2018	sCTX	ELISA	ng/ml	$Oim^{-/-}$	F	120	9	168.28	66.53	WT littermate	F	120	9	12.52	3.14

Animal studies of collagen degradation markers (continue)															
				OI mice						Heathy control mice					
Author, year	Marker	Method	Unit	Genotype	Sex	Age (d)	N	Mean	SD	Control mice	Sex	Age (d)	N	Mean	SD
Matthews 2017	sCTX	ELISA	ng/ml	Oim$^{-/-}$	M	63	8	875.88	181.42	WT littermate	M	63	8	13.02	11.06
Boraschi-Diaz 2017	sCTX	ELISA	ng/ml	Col1a1$^{(Jrt/+)}$	M	28	6	154.70	35.40	WT littermate	M	28	6	36.86	6.32
Boraschi-Diaz 2017	sCTX	ELISA	ng/ml	Col1a1$^{(Jrt/+)}$	F	28	6	70.49	21.48	WT littermate	F	28	6	14.07	2.52
Boraschi-Diaz 2017	sCTX	ELISA	ng/ml	Col1a1$^{(Jrt/+)}$	M	56	8	21.39	10.21	WT littermate	M	56	8	10.38	2.94
Boraschi-Diaz 2017	sCTX	ELISA	ng/ml	Col1a1$^{(Jrt/+)}$	F	56	8	31.64	10.21	WT littermate	F	56	8	12.89	2.91
Boraschi-Diaz 2017	sCTX	ELISA	ng/ml	Col1a1$^{(Jrt/+)}$	M	98	4	19.19	9.28	WT littermate	M	98	4	11.28	2.06
Boraschi-Diaz 2017	sCTX	ELISA	ng/ml	Col1a1$^{(Jrt/+)}$	F	98	4	16.53	4.12	WT littermate	F	98	4	10.69	2.06
Vollersen 2021	sCTX	ELISA	pg/ml	Wnt1$^{+/G177C}$	F	84	5	33.55	14.86	WT littermate	F	84	5	25.05	11.78
Vollersen 2021	sCTX	ELISA	pg/ml	Wnt1$^{G177C/G177C}$	F	84	5	24.86	7.01	WT littermate	F	84	5	25.05	11.78
Vollersen 2021	sCTX	ELISA	pg/ml	Wnt1$^{+/G177C}$	M	84	4	21.50	7.57	WT littermate	M	84	4	17.57	5.70
Vollersen 2021	sCTX	ELISA	pg/ml	Wnt1$^{G177C/G177C}$	M	84	4	27.20	7.94	WT littermate	M	84	4	17.57	5.70
Grafe 2014	sCTX	ELISA	ng/ml	Crtap$^{-/-}$	F	56	14	34.19	5.96	WT littermate	F	56	8	25.58	3.81
Grafe 2014	sCTX	ELISA	ng/ml	Crtap$^{-/-}$	F	112	7	20.82	3.22	WT littermate	F	112	8	14.83	2.97
Greene 2021	sCTX	ELISA	ng/ml	Col1a2$^{(+/G610C)}$	F	140	10	16.78	11.90	WT littermate	F	140	10	13.77	7.14
Uveges 2008	uDPD	ELISA	nmol/mmol Cr	Brtl$^{-/-}$	M	60	7	6.94	1.16	WT littermate	M	60	8	7.31	0.98
Uveges 2008	uDPD	ELISA	nmol/mmol Cr	Brtl$^{-/-}$	M	180	8	3.71	0.53	WT littermate	M	180	7	3.20	0.49

Clinical studies of osteoclast-related parameter from bone histomorphometric analysis												
Author, year	Sex	Type of OI	Parameters	Unit	Age (Y)	OI subjects			Heathy control subjects			
						N	Mean	SD	Reference	N	Mean	SD
Baron 1983	MF	NR	OcS/BS	%	10	9	2.00	1.10	AMG	5	1.10	0.30
Baron 1983	MF	NR	N.Oc/perimeter	no./mm	10	9	0.30	0.16	AMG	5	0.12	0.02
Hryhorovskyi 2015	NR	I	N.Oc/perimeter	no./mm	11	3	3.41	1.53	Glorieux 2000	58	0.32	0.17
Hryhorovskyi 2015	NR	III	N.Oc/perimeter	no./mm	11	3	2.94	2.39	Glorieux 2000	58	0.32	0.17
Iwamoto 2002	M	I	N.Oc/BS	no./mm	58	1	0.22		Recker 2018	48	0.19	0.13
Iwamoto 2002	M	I	Oc.S/BS	%	58	1	0.69		Recker 2018	48	0.34	0.24
Iwamoto 2002	M	I	ES/BS	%	58	1	6.50		Recker 2018	48	1.16	0.63
Leanne Ward 2016	M	VI	N.Oc/perimeter	no./mm	0.71	**1**	0.10		Glorieux 2000	58	0.40	0.20
Leanne Ward 2016	M	VI	OcS/BS	%	0.71	**1**	0.04		Glorieux 2000	58	1.10	0.80
Lui 2022	M	XII	ES/BS	%	6	1	32.15		Glorieux 2000	58	14.80	4.40
Lui 2022	M	XII	Oc.S/BS	%	6	1	3.36		Glorieux 2000	58	1.10	0.75
Rauch 2000	MF	I	ES/BS	%	7.6	32	17.03	6.27	Glorieux 2000	27	15.33	5.06
Rauch 2000	MF	I	Oc.S/BS	%	7.6	32	1.37	0.50	Glorieux 2001	27	1.20	0.87
Rauch 2000	MF	I	N.Oc/BS	no./mm	7.6	32	0.47	0.29	Glorieux 2002	27	0.35	0.18
Rauch 2000	MF	III	ES/BS	%	7.6	11	15.83	-18.16	Glorieux 2003	27	19.57	14.95
Rauch 2000	MF	III	Oc.S/BS	%	7.9	11	2.02	1.41	Glorieux 2004	27	1.20	0.87
Rauch 2000	MF	III	N.Oc/BS	no./mm	7.9	11	0.69	0.36	Glorieux 2005	27	0.35	0.18
Rauch 2000	MF	IV	ES/BS	%	7.5	27	20.37	8.88	Glorieux 2006	27	15.33	5.06
Rauch 2000	MF	IV	Oc.S/BS	%	7.5	27	1.79	1.20	Glorieux 2007	27	1.20	0.87
Rauch 2000	MF	IV	N.Oc/BS	no./mm	7.5	27	0.51	0.29	Glorieux 2008	27	0.35	0.18
Rauch 2002	MF	I, III, IV	OcS/BS	%	8.4	41	1.18	2.30	Glorieux 2009	58	0.32	0.17
Rauch 2002	MF	I, III, IV	ES/BS	%	8.4	44	19.00	1.80	Glorieux 2010	58	1.09	0.58
Ste-Marie 1984 AGD chli	M	NR	ES/BS	%	12	4	5.70	2.50	Glorieux 2000	12	7.60	1.70

**Clinical studies of osteoclast-related parameter from bone histomorphometric analysis (continue)**												
Author, year	Sex	Type of OI	Parameters	Unit	Age (Y)	OI subjects			Heathy control subjects			
						N	Mean	SD	Reference	N	Mean	SD
Ste-Marie 1984 AGD F	F	NR	N.Oc/mm2	no./mm^2	41	4	0.41	0.15	Glorieux 2001	3	0.20	0.09
Ste-Marie 1984 AGD F	F	NR	ES/BS	%	41	4	6.33	3.09	Glorieux 2002	3	3.60	1.10
Ste-Marie 1984 AGD M	M	NR	N.Oc/mm2	no./mm^2	42.3	4	0.21	0.12	Glorieux 2003	3	0.20	0.09
Ste-Marie 1984 AGD M	M	NR	ES/BS	%	42.3	4	4.60	2.82	Glorieux 2004	3	3.60	1.10
Ste-Marie 1984 chli	M	NR	N.Oc/mm2	no./mm^2	12	4	0.45	0.32	Glorieux 2005	12	0.80	0.43
Stürznickel 2021	NR	XX	ES/BS	%	0.46	1	4.61	7.71	AMG	58	1.10	0.80
Stürznickel 2021	NR	XX	N.Oc/B.Pm	no./mm	0.46	1	0.78	0.55	AMG	58	0.40	0.20
Surowieca 2020	MF	I, III, IV, III/IV	N.Oc/B.Pm	no./mm	11.7	8	1.04	0.97	Glorieux 2000	58	0.32	0.17
Webb 2017	M	XIV	ES/BS	%	15	1	2.94		AMG	14	14.90	5.60
Webb 2017	F	XIV	ES/BS	%	24	1	1.55		AMG	14	14.90	5.60
Webb 2017	M	XIV	Oc.S/BS	%	15	1	0.15		AMG	14	1.14	0.74
Webb 2017	F	XIV	Oc.S/BS	%	24	1	0.10		AMG	14	1.14	0.74
Webb 2017	M	XIV	N.Oc/BS	no./mm	15	1	0.04		AMG	14	0.29	0.14
Webb 2017	F	XIV	Oc.S/BS	no./mm	24	1	0.03		AMG	14	0.29	0.14

Animal studies of osteoclast-related parameter from bone histomorphometric analysis

Author, year	Gender	Genotype	Parameters	Organ	Age (wk)	Unit	OI mice			Healthy control mice		
							N	Mean	SD	N	Mean	SD
Uveges 2008	M	Brtl$^{-/-}$	N.Oc	F	8	n/um2	8	6.80	1.03	11	4.68	1.20
Uveges 2008	M	Brtl$^{-/-}$	N.Oc	F	24	n/um2	9	6.37	1.42	13	4.50	1.58
Uveges 2008	M	Brtl$^{-/-}$	N.Oc	F	8		4	215.18	26.07	4	137.31	20.16
Uveges 2008	M	Brtl$^{-/-}$	N.Oc	F	24		6	64.66	5.56	6	44.84	5.56
Uveges 2008	M	Brtl$^{-/-}$	Oc.S/BS	F	8	%	8	14.69	2.85	11	10.77	3.12
Uveges 2008	M	Brtl$^{-/-}$	Oc.S/BS	F	24	%	9	12.30	3.09	13	8.48	2.49
Uveges 2008	NR	Crtap$^{-/-}$	N.Oc	F	12	no/mm	6	10.40	2.55	6	11.75	2.60
Uveges 2008	NR	Crtap$^{-/-}$	Oc.S/BS	F	12	%	6	21.83	6.06	6	18.08	3.85
Chen 2014	M	Col1a1$^{(Jrt/+)}$	N.Oc	F	8	no/mm	6	0.94	0.01	6	0.95	0.02
Joeng 2014	NR	Wnt1$^{sw/sw}$	N.Oc	LV	6	no/mm	5	6.85	1.14	5	5.27	1.00
Joeng 2014	NR	Wnt1$^{sw/sw}$	Oc.S/BS	LV	6	%	5	12.89	2.65	5	10.20	1.23
Chen 2014	M	Col1a1$^{(Jrt/+)}$	Oc.S/BS	F	8	ratio	6	0.05	0.01	6	0.05	0.01
Gruenwald 2014	M	Sc65$^{-/-}$	Oc.S/BS	T	10	%	6	0.75	1.26	6	1.06	1.34
Grafe 2014	F	Crtap$^{-/-}$	N.Oc	LV	16	no/mm	6	4.83	0.87	6	3.23	0.82
Grafe 2015	F	Crtap$^{-/-}$	N.Oc	LV	7	N/BS	6	4.93	0.48	6	3.85	0.44
Grafe 2015	F	Crtap$^{-/-}$	N.Oc	LV	12	N/BS	6	9.65	3.55	6	6.09	0.60
Grafe 2015	F	Crtap$^{-/-}$	Oc.S/BS	LV	7	%	6	18.87	1.62	6	15.97	2.88
Grafe 2015	F	Crtap$^{-/-}$	Oc.S/BS	LV	12	%	6	29.80	5.04	6	23.47	1.56
Patoine 2017	M	Bril$^{-/-}$	N.Oc	F	9	no/mm	4	3.25	1.02	4	3.03	0.52
Patoine 2017	M	Bril$^{-/-}$	Oc.S/BS	F	9	%	4	7.55	2.48	4	6.80	1.56
Patoine 2017	M	Bril$^{-/-}$	ES/BS	F	9	%	4	36.80	4.40	4	28.50	7.20
Jeong 2018	M	Col1a2$^{(+/G610C)}$	N.Oc	F	16	no/mm	4	4.09	1.65	6	2.58	0.81

**Animal studies of osteoclast-related parameter from bone histomorphometric analysis (continue)**												
Author, year	Gender	Genotype	Parameters	Organ	Age (wk)	Unit	OI mice			Healthy control mice		
							N	Mean	SD	N	Mean	SD
Jeong 2018	F	Col1a2 $^{(+/G610C)}$	N.Oc	F	16	no/mm	4	5.47	1.02	7	5.53	1.26
Jeong 2018	M	Col1a2 $^{(+/G610C)}$	Oc.S/BS	F	16	%	4	9.21	3.31	6	6.98	2.27
Jeong 2018	F	Col1a2 $^{(+/G610C)}$	Oc.S/BS	F	16	%	4	13.06	2.44	7	14.15	2.68
Jeong 2018	M	Oim$^{-/-}$	N.Oc	F	16	no/mm	3	5.26	1.63	6	2.58	0.81
Jeong 2018	F	Oim$^{-/-}$	N.Oc	F	16	no/mm	3	7.37	1.30	7	5.53	1.26
Jeong 2018	M	Oim$^{-/-}$	Oc.S/BS	F	16	%	3	12.55	3.72	6	6.98	2.27
Jeong 2018	F	Oim$^{-/-}$	Oc.S/BS	F	16	%	3	18.26	1.63	7	14.15	2.68
Zimmerman 2018	M	Oim$^{-/-}$	N.Oc	LV	12	no/mm	8	1.33	1.33	8	2.32	0.96
Zimmerman 2018	M	Oim$^{-/-}$	Oc.S/BS	LV	12	%	8	2.16	2.50	8	4.96	1.85
Wang 2019	NR	Wnt1 $^{prrx1-/-}$	N.Oc	T	6	no/mm	6	5.71	2.17	6	5.17	0.97
Wang 2019	NR	Wnt1 $^{prrx1-/-}$	N.Oc	T	12	no/mm	6	4.69	0.70	6	3.52	0.56
Wang 2019	NR	Wnt1 $^{prrx1-/-}$	ES/BS	T	6	no/mm	6	4.11	1.25	6	3.22	1.06
Wang 2019	NR	Wnt1 $^{prrx1-/-}$	ES/BS	T	12	no/mm	6	5.73	1.19	6	3.78	0.70
Greene 2021	F	Col1a2 $^{(+/G610C)}$	N.Oc	F	20	ratio	5	5.61	1.29	8	2.93	0.42
Vollersen 2021	F	Wnt1$^{+/G177C}$	N.Oc	F	4	no/mm	3	3.47	0.74	5	2.62	0.51
Vollersen 2021	F	Wnt1$^{+/G177C}$	N.Oc	F	12	no/mm	6	3.21	0.79	5	2.40	0.72
Vollersen 2021	F	Wnt1$^{+/G177C}$	N.Oc	F	24	no/mm	5	3.08	1.14	3	5.15	0.12
Vollersen 2021	M	Wnt1$^{+/G177C}$	N.Oc	F	4	no/mm	5	3.08	0.66	4	3.24	0.63
Vollersen 2021	M	Wnt1$^{+/G177C}$	N.Oc	F	12	no/mm	6	3.37	0.54	6	2.47	1.27
Vollersen 2021	M	Wnt1$^{+/G177C}$	N.Oc	F	24	no/mm	4	3.60	1.30	5	2.91	1.33
Vollersen 2021	F	Wnt1$^{(G177C/G177C)}$	N.Oc	F	4	no/mm	6	4.20	0.94	5	2.62	0.51
Vollersen 2021	F	Wnt1$^{(G177C/G177C)}$	N.Oc	F	12	no/mm	5	2.75	0.82	5	2.40	0.72

Animal studies of osteoclast-related parameter from bone histomorphometric analysis (continue)												
Author, year	Gender	Genotype	Parameters	Organ	Age (wk)	Unit	OI mice			Healthy control mice		
							N	Mean	SD	N	Mean	SD
Vollersen 2021	F	Wnt1$^{(G177C/G177C)}$	N.Oc	F	24	no/mm	5	3.79	0.71	3	5.15	0.12
Vollersen 2021	M	Wnt1$^{(G177C/G177C)}$	N.Oc	F	4	no/mm	6	3.79	1.48	4	3.24	0.63
Vollersen 2021	M	Wnt1$^{(G177C/G177C)}$	N.Oc	F	12	no/mm	5	5.10	0.56	6	2.47	1.27
Vollersen 2021	M	Wnt1$^{(G177C/G177C)}$	N.Oc	F	24	no/mm	8	3.41	1.15	5	2.91	1.33

Supplemental Figure 1: Subgroup analysis of collagen degradation markers from clinical data by sex of OI patients

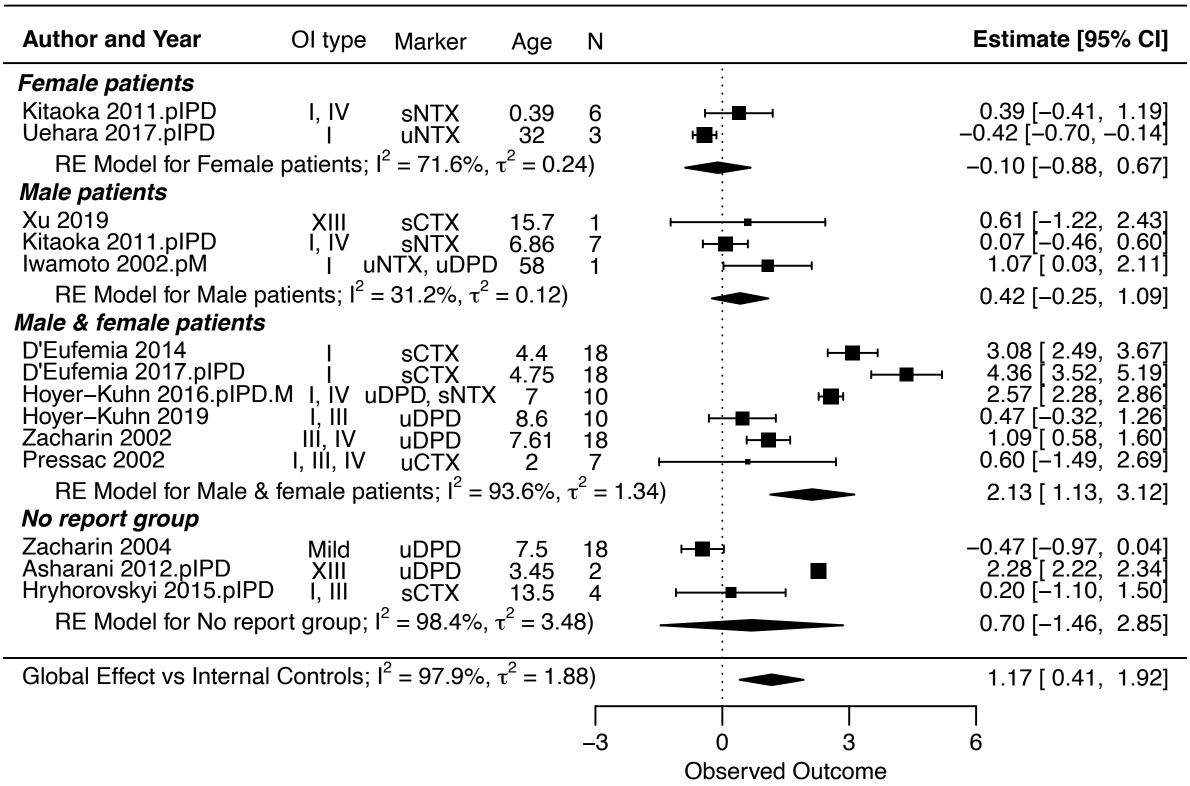

Forest plots of subgroup analysis by sex of OI patients. Indicated are the included studies, the OI type for reported patients for markers measured in the study; age of patients, and the number of patients. The standardized mean differences with 95% confidence interval (CI) for individual studies are depicted as squares/lines, the square size is proportional to the study weight. Diamonds/bands are global effect size and CI. Positive difference reflects higher values in OI. The heterogeneity statistics I^2 and τ^2 are reported.

Supplemental Figure 2. Subgroup analysis of collagen degradation markers from clinical data by type of markers

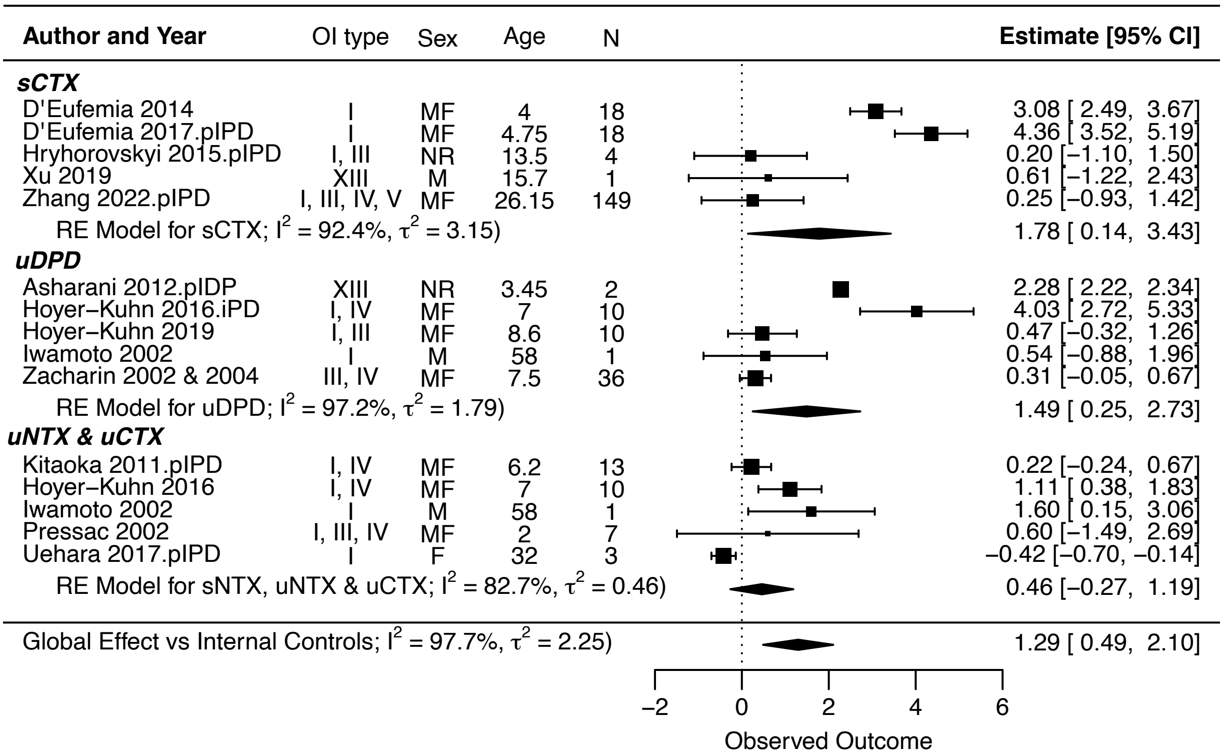

Forest plots of subgroup analysis by type of reported marker. Indicated are the included studies, the OI type for reported patients for markers measured in the study; sex of patients as male (M), female (F), mixed group of male and female (MF) and not reported (NR), age of patients, and the number of patients. The standardized mean differences with 95% confidence interval (CI) for individual studies are depicted as squares/lines, the square size is proportional to the study weight. Diamonds/bands are global effect size and CI. Positive difference reflects higher values in OI. The heterogeneity statistics I^2 and τ^2 are reported.

Supplemental Figure 3: Effect of biological factors on histomorphometric osteoclast parameters in OI mice.

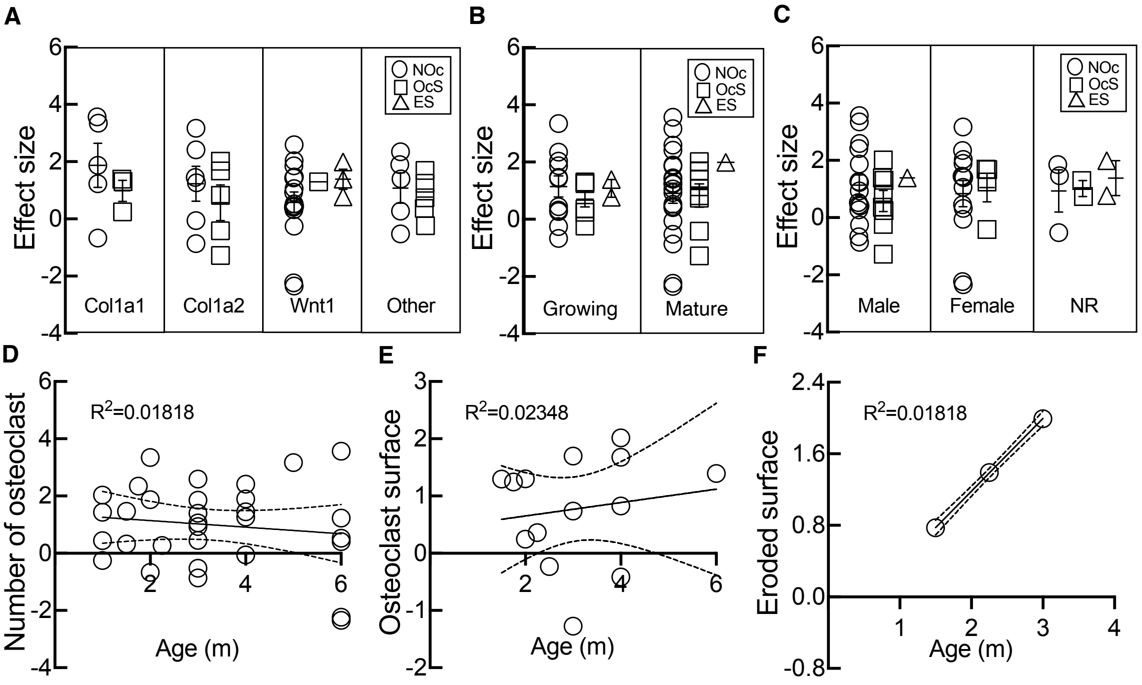

The effect sizes from individual outcomes for osteoclast numbers (NOc, circles), osteoclast surface (OcS, squares) or eroded surface (ES, triangles) reported in each study were separated by **(A)** the underlying mutation in *Col1a1*, *Col1a2, Wnt1* or other genes, **(B)** age grouped as growing (younger than 2 months) and mature (older than 3 months), and **(C)** reported sex of OI animal. Shown are means ±SEM, no statistical significance by one-way ANOVA. Linear regression analysis for age effect on osteoclast numbers **(D)**, osteoclast surface **(E)** and eroded surface **(F).** R^2 indicates goodness of fit.

Supplemental Figure 4: Publication bias, heterogeneity and sensitivity analyses for collagen degradation markers from clinical and animal studies

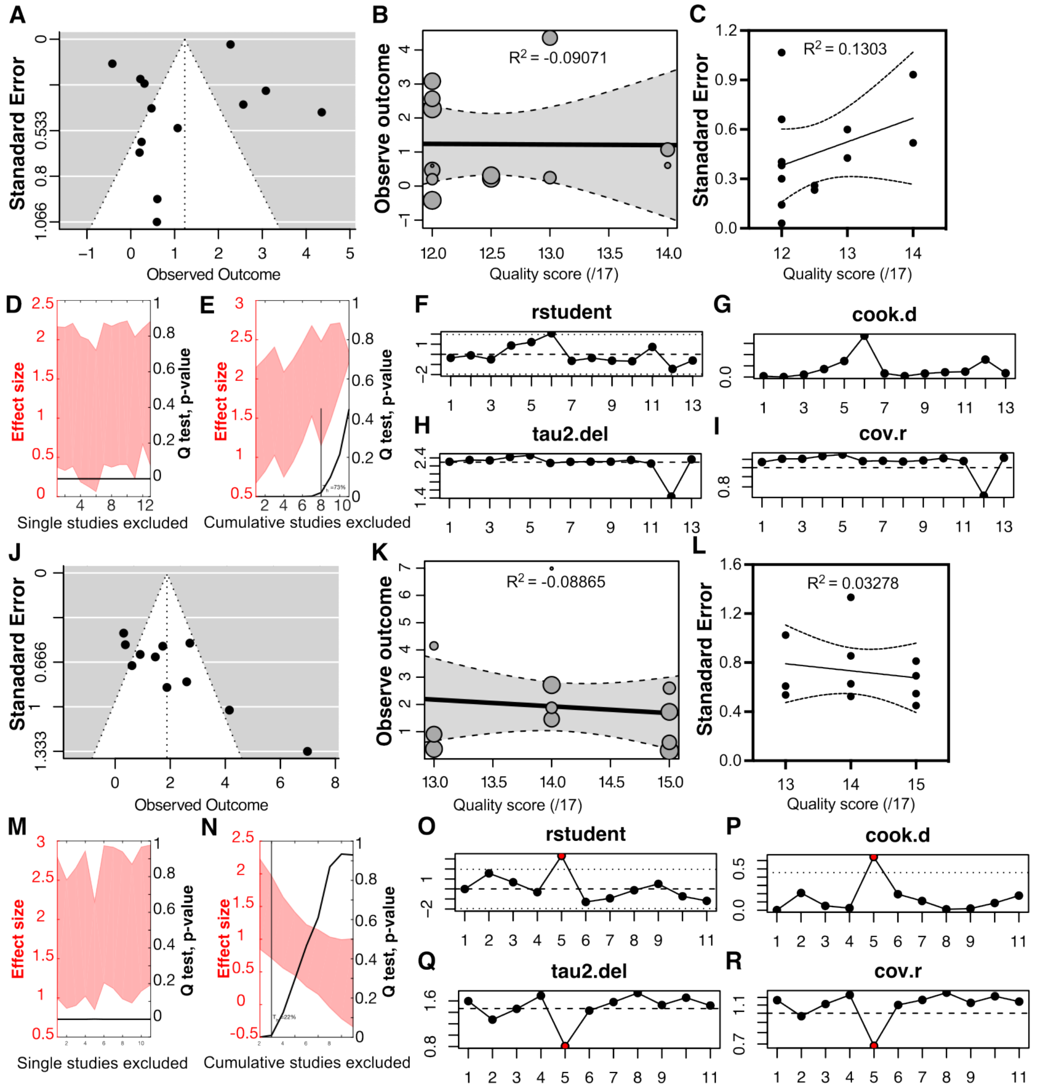

Analyses for collagen degradation markers from clinical and animal studies (A-I) and animal studies (J-R). Publication bias and quality of studies were assessed using funnel plot (A, J), meta-regression of observed outcome with quality score (B, K) and linear-regression of standard error with quality score (C, L). Heterogeneity was analyzed using single study exclusion (D, M) and cumulative study exclusion (E, N). Red area: 95% CI for the global effect size (left axis); line: p-value of Q test (right axis). Influential studies were analyzed using standardized residual (rstudent) (F, O), cook's distances (cook.d) (G, P), leave-one-out amount of heterogeneity (tau2.del) (H, Q) and covariance ratio (cov. r) (I, R).
